# Immunolocalization of hordein synthesis and transport in developing barley endosperm

**DOI:** 10.1002/pld3.591

**Published:** 2024-09-05

**Authors:** Gregory Tanner, Allison van de Meene, Anthony Bacic

**Affiliations:** ^1^ School of Biosciences The University of Melbourne Melbourne Victoria Australia; ^2^ Ian Holmes Imaging Centre, Bio21 Institute The University of Melbourne Melbourne Victoria Australia; ^3^ La Trobe Institute for Sustainable Agriculture & Food Department of Animal, Plant and Soil Sciences, School of Agriculture, Biomedicine and Environment La Trobe University Bundoora Victoria Australia

**Keywords:** anthesis, developing barley endosperm, hordein, immunoelectron microscopy, immunofluorescent microscopy, immunolocalization, LTP1, serpin Z4

## Abstract

The spatial accumulation of hordeins in the developing endosperm of barley grains was examined by immunofluorescence microscopy (immunolight microscopy [iLM]) and immunoelectron microscopy (iEM) to establish the timing and subcellular pattern of hordein synthesis and deposition. The pattern seen for hordeins was compared to other abundant grain proteins, such as serpin Z4 and lipid transfer protein 1 (LTP1). Hordein accumulates throughout grain development, from 6 to 37 days post‐anthesis (DPA). In contrast, serpin Z4 was present at 6 DPA, but the greatest synthesis and accumulation occurred during the middle of seed development, from 15 to 30 DPA. LTP1 accumulated later in seed development, from 15 to 30 DPA. Hordeins accumulated within the lumen of the endoplasmic reticulum (ER), were exocytosed from the ER membrane, and accumulated in protein bodies, which then fused either with the protein storage vacuoles or with other protein bodies, which also later fused with the protein storage vacuoles. iEM showed hordein, and LTP1 appeared not to traverse the Golgi apparatus (GA). Hordein, LTP1, and serpin Z4 colocalized to the same protein bodies and were co‐transported to the protein storage vacuole in the same protein bodies. It is likely that this represents a general transport mechanism common to storage proteins in developing grains.

## 1 INTRODUCTION

Prolamins, so‐called as they are rich in proline (P) and glutamine (Q) residues, are a collective name given to gluten, the alcohol‐soluble, water‐insoluble storage proteins found in the grains of wheat (gliadin and glutenin), barley (hordein), rye (secalins), and oats (avenin). The barley hordeins account for 10% of soluble protein and consist of four closely related protein families: the D‐hordeins, a 105‐kDa protein family coded for by a single gene (Gu et al., 2003) with up to five post‐translationally modified isoforms (Tanner et al., 2014); the C‐hordeins, which are 55‐ and 65‐kDa sulfur‐poor proteins, coded for by 20–30 genes (Entwistle, 1988; Shewry, Bunce, et al., 1985); the B‐hordeins, which are a group of sulfur‐rich proteins running at 50 kDa, coded for by at least 13 genes, with at least two protein sub‐families, the B1‐ and B3‐hordeins (Anderson, 2013; Kreis et al., 1983); and the sulfur‐rich γ‐hordeins consisting of 45 kDa ( γ‐1‐hor, 1 gene), 40 kDa ( γ‐2‐hor, produced from the γ‐1‐protein by post‐translational deletion of 30 amino‐acids), and 35 kDa ( γ‐3‐hor, 1 gene) proteins (Cameron‐Mills & Brandt, 1988; Shewry, Kreis, et al., 1985). The four protein families are coded for by Hor‐1 (C‐hordein), Hor‐2 (B‐hordein), Hor‐3 (D‐hordein), and Hor‐4 ( γ‐hor) loci, located on barley chromosome 1H (Kreis & Shewry, 1992). The B‐hordeins account for 70%–90% of total hordeins; the C‐hordeins form 10% to 30% of the hordein fraction; the γ‐hordeins and the D‐hordeins are minor components accounting for 1%–2% and 2%–4%, respectively, of the hordeins (Shewry, Kreis, et al., 1985). Hordeins accumulate in the starchy endosperm of developing barley grains during grain filling. Hordein synthesis proceeds linearly from approximately 10 to 30 days post‐anthesis (DPA) (Brandt, 1976; Sorensen et al., 1989; Tanner et al., 2019). Hordeins do not appear to have any catalytic role and function only as a protein reserve for germination; however, particular hordeins may have a role as molecular chaperones. It has been postulated that γ‐3‐hordein is involved in folding the other hordeins into a transport‐competent complex (Rechinger, Simpson, et al., 1993). Prolamins may also be involved in the formation of protein bodies. The expression of prolamins (the N‐terminal half of the γ‐zein transgene) is involved with protein body formation and induces the formation of prolamin‐containing endoplasmic reticulum (ER)‐derived protein bodies in Arabidopsis (Arcalis et al., 2019).

In addition to examining the location and deposition of hordeins, we examined the location of two other dominant storage proteins, serpin Z4 and lipid transfer protein 1 (LTP1), both accounting for approximately 5% of total grain protein in mature barley endosperm. Serpin Z4 is coded for by a single gene (Brandt et al., 1990) and is synthesized without a signal peptide; mRNA is associated with the ER (Brandt et al., 1990). Serpin Z4 accumulates as three closely related isoforms produced from the same parent protein by post‐translational proteolytic modification, and all are collectively known as serpin Z (previously protein Z) (Roberts et al., 2003). Serpin Z is postulated to serve a protective function for grains by inhibiting the gut proteases of insect predators (Cohen & Fluhr, 2018) by acting as a suicide inhibitor of serine‐like proteases such as trypsin and chymotrypsin (Ferreira et al., 2023). The inhibited form of serpin Z4 may act as a β‐amylase‐specific molecular chaperone (Cohen & Fluhr, 2018). LTP1 is coded for by a single gene (Mundy & Rogers, 1986; Skriver et al., 1992). The protein consists of 91 amino acids with a mass of 9.7 kDa. The mature protein is involved in the transfer of phosphatidylcholine and other lipids (Breu et al., 1989; Edqvist et al., 2018).

It is confounding to imagine how considerable quantities of water‐insoluble prolamin can accumulate within the aqueous environment of a cell, but these proteins do accumulate inside the cytoplasm and within protein bodies. Plant vacuoles form the distal termini of a transport process that carries storage proteins from the ER. There are two main forms of plant vacuoles: the lytic vacuole in vegetative tissues and the protein storage vacuole in developing grains (Cui et al., 2020). In developing cereal grains, protein transport proceeds from the site of synthesis in the ER lumen, sometimes transiting the Golgi apparatus (GA), depending on the nature of the protein, and then to protein bodies, which ultimately fuse with the vacuole (Shimada et al., 2018; Tan et al., 2019). The process of protein body initiation from ER is continuous throughout grain maturation (Moore et al., 2016; Roustan et al., 2020). Protein disulfide isomerase (PDI) has been shown to be involved in protein body formation in barley, is associated with ER and protein bodies, and also appears to be involved in protein folding in the ER (Roustan et al., 2018).

In developing cereal grains, prolamins are deposited in different organelles depending on the species. In tropical grains (maize and rice), prolamins accumulate within the lumen of the ER, whereas in wheat, oats, and barley, the prolamins are destined for transport and accumulation in the protein storage vacuoles (Shewry & Halford, 2002). In wheat, prolamins are either transported via the GA to protein bodies and then to the vacuole or accumulate directly within the lumen of the ER (Tosi et al., 2009).

In barley, hordeins are initially associated with the ER (Miflin et al., 1981), and biochemical studies show that they are coordinately expressed on the polyribosomes of the ER in developing starchy endosperm cells. The ~20 amino‐acid N‐terminal transit peptides are removed from hordeins during co‐translational processing and transport into the ER lumen (Cameron‐Mills et al., 1978; Cameron‐Mills & Madrid, 1989). Hordeins are then exocytosed from the ER lumen to the cytoplasm (Cameron‐Mills et al., 1978; van de Meene et al., 2017) and then accumulate in protein bodies, which fuse with the protein storage vacuole (Rechinger, Simpson, et al., 1993; Roustan et al., 2018, 2020). We confirm this sequence by immunoelectron microscopy (iEM) here. Several specific proteins involved in endosomal sorting complexes required for transport (ESCRT)‐III have been identified in developing barley endosperm (Hilscher et al., 2016; Ibl, 2019). Protein sorting into protein bodies during barley endosperm development is putatively regulated by two cytoskeleton proteins (Roustan et al., 2020).

The involvement of the GA in glycan decoration, sorting, and intracellular transport of plant proteins to different destinations is well established (Ebert et al., 2018; Strasser et al., 2021). However, the role of the GA in the transport of hordeins in barley is not clear. The pathway taken by hordeins, from protein body to vacuole, is reported to either bypass the GA (Ibl et al., 2014; Rechinger, Simpson, et al., 1993) or traverse the GA, but clear electron microscopical evidence of this was lacking (Bethke & Jones, 2000; Møgelsvang & Simpson, 1998). The pathway taken by prolamins differs between species; for example, wheat gliadin has been clearly localized to the GA (Kim et al., 1988); however, a second gliadin transport route in wheat that bypasses the GA has also been reported (Galili et al., 1993; Levanony et al., 1992; Tosi et al., 2009).

Many individual steps in hordein intracellular transport and accumulation have been postulated, and biochemical evidence is often obtained by extrapolation from other species. Here, we present a more comprehensive picture of hordein location and transport in developing barley endosperm cells, derived by immunolight microscopy (iLM) and iEM immunolocalization. We examine the intracellular location and hence deduce the pathway taken by hordeins during the development of the starchy endosperm of barley. We also examine the location of two other abundant storage proteins, serpin Z4 and LTP1. The transport of hordein protein from the ER to protein bodies and eventually to the vacuoles is a complex process that involves the coordinated action of multiple proteins and vesicle transport pathways. Defining this process is the first step required for manipulating the nutritional quality of grain storage proteins.

## 2 METHODS

### 2.1 Plant growth

**FIGURE 1:**
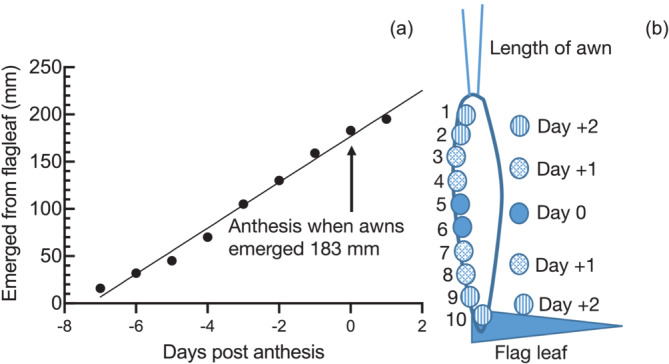
(a, b) Development of barley cv Sloop grains in the head. Anthesis occurs 8 weeks after germination and spreads progressively, moving outwards from the center two grains and lastly occurring in the two top and bottom grains in the head 2 days post‐anthesis. These figures are for cv Sloop grown under stated conditions. The relationship between awn length and anthesis differs for other cultivars and conditions.

Barley cv Sloop was obtained from the Australian Winter Cereals Collection and germinated at weekly intervals in a 50:50 (v/v) mixture of soil (Debco Seed Raising Mix, Tyabb, Victoria) and perlite, three plants per 20 cm pot, and grown at a constant temperature of 19–24°C, under ambient light with daylight extension to 12 h by 1500 W of halogen lights at 400 μE. Plants were watered with a balanced nutrient solution (250 ml per pot of 2.5 g/L Aquasol, Yates Australia Padstow) once every week. Supplemental lights were supplied in the morning and evening, so the daylength was maintained at 12 h from March to October. In Australia, barley is a winter crop. In the summer, outside these months when daylength exceeded 13 h, flowering was not synchronous and seed set was poor, as the heads bolted early. Anthesis normally began about 8 weeks after germination and was determined by daily inspection before 10 a.m. and was taken as the first day that that pollen was freely released by an anther from the center grains. The heads were labeled with anthesis date. In practice, this was when the head was about half extended from the flag leaf; under these conditions, Sloop flowers first in the middle two grains of the head, and then the flowering progressively spreads up and down the head each day after anthesis (Figure 1). The center two grains were harvested from duplicate heads on the indicated DPA.

### 2.2 Standard fixation and embedding

The hull was removed, and 1‐mm longitudinal and transverse hand sections were cut with a scalpel from duplicate grains at 6, 8, 10, 15, 20, 25, 30, and 37 DPA, immediately immersed in glutaraldehyde‐formaldehyde (GAF) fixative (.1% [w/v] glutaraldehyde, 4% [w/v] formaldehyde in phosphate‐buffered saline [PBS]) incubated under vacuum for 1 h and then overnight at 4°C, washed in PBS for 1 min, then dehydrated in an ethanol (EtOH) series (1 h in 10%, 30%, 50%, 70%, and 90% v/v EtOH and three changes of 100% [v/v] EtOH) followed by LR White (LRW) resin (ProSciTech) series for 1 day each (5%, 10%, 25%, 50%, and 75% v/v LRW in ethanol and three changes of 100% [v/v] LRW), and polymerized overnight at 53°C (Wilson & Bacic, 2012). Unless otherwise noted, iEM images were obtained from standard fixation.

### 2.3 High pressure freezing/freeze substitution (HPF)

With the above procedure, the onset of fixation takes minutes, depending on the depth and rate of penetration of the fixative. For immediate fixation, thin (~200 μm) cross sections were cut by hand from fresh grains at the indicated DPA and immediately subjected to HPF as follows: Sections were placed on a 200‐μm membrane carrier, sealed, and plunged into liquid N at elevated pressure and frozen using a Leica EMPACT2 HPF. The frozen tissue samples were subjected to freeze substitution solvent exchange at low temperature (−85°C) with .2% (w/v) uranyl acetate in acetone for 72 h. The samples were then slowly warmed to room temperature (RT) over 24 h, followed by a wash in acetone, then a 1:1 solution of acetone:ethanol, and then 100% ethanol twice. The samples were infiltrated and embedded with LRW resin as above.

### 2.4 Antibody design

Family‐specific hordein antibodies have been reported (Rechinger, Bougri, & Cameron‐Mills, 1993; Rechinger, Simpson, et al., 1993; Ullrich et al., 1986), but due to intellectual property (IP) limitations, we were unable to access these antibodies. From PILEUP analysis of combined hordein sequences, eight unique peptides (P1–P8) were designed to identify the B‐, C‐, or D‐hordein families (Figure S1), and monoclonal antibodies raised in mice to the indicated synthetic peptides (ABMART, Shanghai, China). Unfortunately, no monoclonal antibodies displayed the intended family specificity. Four of the ascites clones (raised to P1, P3, P4, and P5) did not produce useful antibodies, and the remaining ascites fluids reacted with all hordein families (Table S1). It was thought that this may be due to a high content of P and Q residues, so additional monoclonals were raised to modify family‐specific peptides with reduced P and Q residues (Table S1). Unfortunately, these second round monoclonals also identified all hordein families. The reason for this lack of familial specificity is unknown but could be related to the use of wheat rations fed to the mice. Preliminary clonal screening may have mistakenly identified preexisting antibodies to dietary gluten proteins that reacted to the gluten‐like peptides used to screen monoclonals. However, monoclonals designated B4 raised against synthetic peptide P2 (C‐TQQQLQQEQVGQ) and 23‐3 raised earlier against γ‐hordein peptide (C‐SFLRPHISQQNS) (Tanner et al., 2016) were excellent general anti‐hordein antibodies and gave the same results as rabbit anti‐gliadin‐horseradish peroxidase (HRP) (Sigma) on western blots (Tanner et al., 2013). In all results, mouse anti‐hordein 23‐3 was used unless otherwise specified.

Rabbit polyclonal anti‐peptide antibodies to LTP1 (lab designation V6177) and serpin Z4 (V6175) were produced and purified by immunochromatography by GenScript (Piscataway, NJ, USA). Antibodies from antigenic peptides identified within LTP1 (P07597.1; D33LHNQAQSSGDRQT46) and serpin Z4 (P06293.2; R258LSTEPEFIENHIP271) were raised in rabbits fed on alfalfa rations (Tanner et al., 2019).

### 2.5 Sectioning and staining by immunofluorescence by light microscopy (LM) and iLM

Sections (1 μm) were cut with glass knives from resin‐embedded tissues, floated on a water bath, transferred to glass slides, dried at 60°C, and stained with .5% (w/v) Toluidine Blue O, then washed and examined under brightfield (LM), or blocked in .5% (w/v) bovine serum albumin (BSA) in PBS Tween (PBST) for 1 h (iLM). Blocked sections were incubated in the indicated 1/100 primary antibody in PBST (1 h), washed thrice in PBST, and incubated with 1/100 secondary antibody (Invitrogen donkey anti‐mouse IgG‐Alexa Fluor 568 (Invitrogen) or goat anti‐rabbit IgG‐Alexa Fluor 568 (Abcam)) in PBST (1 h), washed five times in PBST, covered in 50% (v/v) glycerol, and sealed under a cover slip. Slides were examined for fluorescence in a Leica DM6000 microscope at 40× or 63×, with an excitation wavelength of 540–550 nm and an emission wavelength of 567–640 nm unless otherwise specified. Sections from duplicate grains gave similar results. Control slides with only secondary antibodies did not generate a fluorescent signal under excitation (Figure S11A,B).

### 2.6 Sectioning and staining by iEM

Sections (90 nm) were cut as above and transferred to Formvar‐coated gold grids, air dried, and blocked by floating on 50 μl drops of 3% (w/v) BSA in PBST for 30 min and washed with PBS and then distilled water and air dried. Grids were floated on 50 μl drops of 1/100 primary antibody in PBST overnight at 4°C, then washed thrice in 3% (w/v) BSA in PBS, and incubated by floating on 50 μl drops of 1/100 secondary antibody for 2 h at RT. Grids were washed in PBS and then distilled water and air dried. Secondary antibodies were either goat anti‐mouse‐IgG‐18 nm gold (Jackson ImmunoResearch Affinity Pure) or goat anti‐rabbit‐IgG‐10 nm gold (Sigma). Unfortunately, partway through this investigation, these reagents had to be renewed, and the originals were unavailable. Unfortunately, only the reverse designation was available, that is, goat anti‐mouse IgG‐10 nm gold (Sigma) or donkey anti‐rabbit IgG‐18 nm gold (Abcam). To avoid confusion, all secondary antibodies are identified in figure legends. Grids made from duplicate grains gave similar results. Control grids with only the second antibody did not produce any significant signal (Figures S12–S15). Grids were post‐stained with 2% (w/v) uranyl acetate for 15 min, washed, dried, and stained with .5% (w/v) triple lead citrate for 1 min, washed, and dried (Wilson & Bacic, 2012), and examined with a FEI Tecnai Spirit TEM (Thermo Fisher Scientific).

## 3 RESULTS

### 3.1 Anthesis in barley cv Sloop

In cv Sloop, anthesis commenced 8 weeks after germination, firstly in the two grains at the center of the head and radiating outwards (Figure 1). There was a linear relationship between the initiation of anthesis in the center two grains and the extension of the awns from the head. Anthesis in the center two grains occurred when the awns had extended by 183 mm and spread progressively as a wave moved outwards from the center two grains. Two days after commencement, anthesis was complete in the outermost two grains at the top and bottom of the head.

### 3.2 Temporal accumulation of hordeins, LTP1, and serpin Z4

Hordeins were synthesized and accumulated early after anthesis in a linear fashion, starting at about 6 DPA and reaching a maximum around 30 DPA (Tanner et al., 2019; Figures S2 and S3C). The accumulation and localization of two additional abundant proteins were also studied. Serpin Z4 accumulates from the middle of seed development and was detectable by western blot from 6 DPA, but unlike hordeins, the bulk of serpin Z4 accumulation occurred only after mid‐development, beyond 15 DPA, and reached a maximum intensity at 30 DPA and is therefore a mid‐period accumulator (Figure S3A). Serpin Z4 was present as three isoforms on western blots: serpin Z7 (47.3 kDa), native serpin Z4 (44.3 kDa), and hydrolyzed serpin Z4 minus 4 kDa active loop (41.6 kDa) (Roberts et al., 2003). The anti‐serpin Z4 antibody identified the three isoforms of serpin Z4 on western blots at the expected molecular weights. The remaining protein, LTP1, accumulated late in seed development and was not detectable on western blots until 15 DPA and accumulated to a maximum at about 30 DPA (Figure S3B). The anti‐LTP antibody identified a single band on western blots at the expected molecular weight of 9 kDa.

### 3.3 Development of starchy endosperm

**FIGURE 2:**
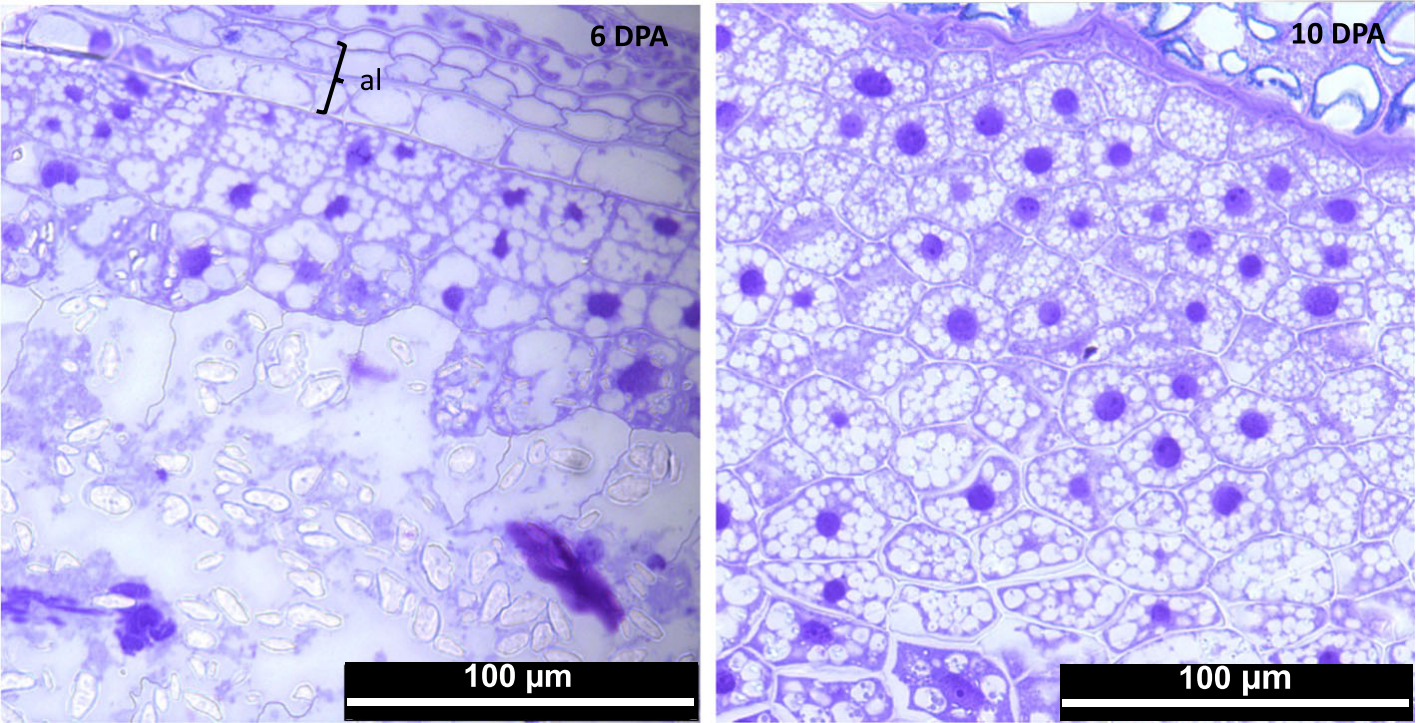
Light microscopy of fully cellularized endosperm cells in the developing grain, showing the aleurone/sub‐aleurone and the peripheral starchy endosperm cells. One‐micron sections were cut from barley cv Sloop grains and stained with toluidine blue at 6 and 10 days post‐anthesis (DPA). The plane of section of the 6 DPA sample is slightly oblique and catches some of the maternal (pericarp/testa) cells external to the aleurone. Amyloplast/vacuole development radiates out from the peripheral endosperm towards the inside of the grain. Cells are filled with starch granules. Immunoelectron microscopy was conducted on the maturing peripheral starchy endosperm cells below the area shown in these figures. Scale bars are all 100 μm.

**FIGURE 3:**
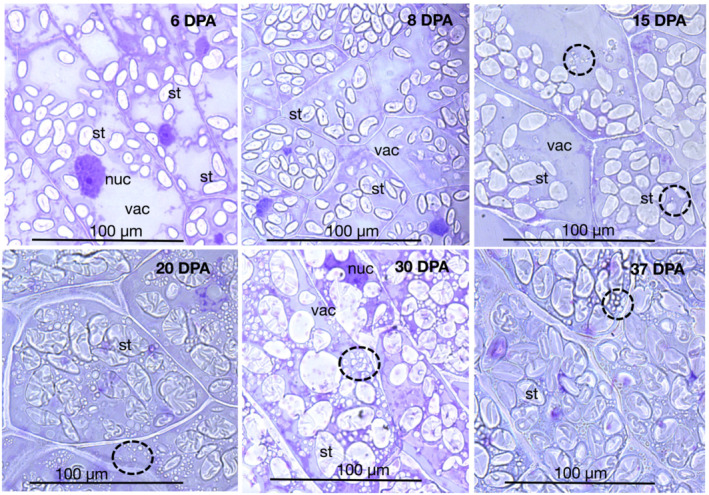
Light microscopy of barley cv Sloop peripheral starchy endosperm cells. Toluidine blue stained 1‐μm sections at 6, 8, 15, 20, 30, and 37 days post‐anthesis (DPA), showing amyloplasts containing starch granules (*st*), nucleus (*nuc*), vacuole (*vac*) at all stages of development, and small vacuoles consisting of single and multiple protein bodies (circled) at later stages (15–37 DPA). Scale bars are all 100 μm.

Barley endosperm is formed from a single triploid endosperm cell, which repeatedly undergoes mitosis without cytokinesis to form a multinuclear syncytium with nuclei distributed around the peripheral layer of the central cell wall surrounding a large central vacuole. Anticlinal walls then form between adjacent syncytial nuclei, which subsequently divide to form periclinal walls. Cellularization of the endosperm proceeds with approximately five to six rounds of successive anticlinal and periclinal wall formation. Following cellularization, during the differentiation phase, the aleurone layer forms by division of the peripheral layer of endosperm cells (Wilson et al., 2006). Barley cv Sloop grains contained two to three layers of aleurone cells, immediately below the testa–pericarp (maternal tissue), which acts as a limiting boundary of the grain (Figure 2). Following cellularization, the endosperm cells undergo differentiation and maturation, which includes the starchy endosperm cells accumulating starch and protein (Figures 2 and 3) and the deposition of cell wall polysaccharides (Wilson et al., 2006).

**FIGURE 4:**
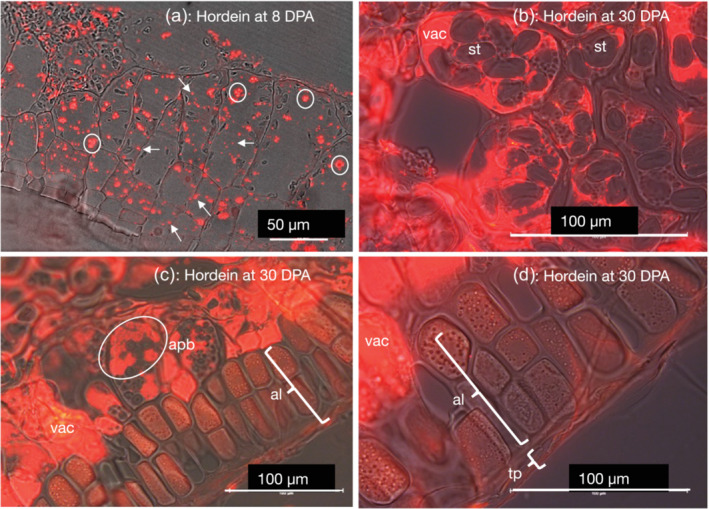
Hordein distribution in the aleurone/sub‐aleurone starchy endosperm cells of barley cv Sloop by immunolight microscopy. (a) In young tissue, at 8 days post‐anthesis (DPA), hordein was in isolated protein bodies (white arrows) in the cytoplasm. Multiple protein bodies were observed fusing (circled). Scale: 50 μm. (b) At 30 DPA, hordein‐containing protein bodies had fused with the central vacuole (*vac*), and amyloplasts containing starch granules (*st*) were silhouetted in front of the vacuole. Scale bar: 100 μm. (c) Hordein accumulation was not observed in aleurone (*al*). Hordein was in either the central vacuole (*vac*) or accumulating protein bodies within small vacuoles (circled, *apb*) of starchy endosperm cells. Scale bar: 100 μm. (d) Higher magnification of the epidermis in (c). Hordein accumulation was not observed in either the aleurone (*al*) or testa/pericarp (*tp*), which had collapsed due to desiccation. Accumulating hordein was seen in the vacuoles of neighboring starchy endosperm cells (*vac*). Scale: 100 μm. Primary antibody (Ab) was mouse anti‐hordein 23‐3; similar results were obtained with mouse anti‐hordein‐B4. Secondary Ab was anti‐mouse‐Alexa Fluor 568.

As the grain progressed from the differentiation phase to the maturation phase, the appearance of these peripheral starchy endosperm cells changed from 6 to 37 DPA (Figure 3). First, starch grains began to form in developing amyloplasts, which became more numerous and progressively filled the interior of the cell by 15 DPA. From 15 DPA onwards, the starch granules began to exhibit a folded appearance. Vacuoles were often partially obscured by numerous amyloplasts that lay above the vacuole in sections. From 20 to 30 DPA, in addition to large amyloplasts, numerous small vesicles began to accumulate in the interior of cells. The similarity in size and distribution confirmed that they were the protein bodies seen by immunofluorescence below (see Figure 4). By 30 DPA, the testa–pericarp (maternal tissue) had collapsed due to desiccation and nutrient resorption, and by 37 DPA, the cell volume (cytoplasm) had decreased.

### 3.4 Spatial accumulation of hordeins, LTP1, and serpin Z4

Hordein distribution in the starchy endosperm, but not in the aleurone, was clearly seen by immunofluorescence microscopy using an anti‐hordein‐B4 antibody (Figure 4). In peripheral endosperm cells at 8 DPA, isolated protein bodies and chains of protein bodies containing hordein could be seen. Multiple protein bodies were seen fusing with each other to form larger protein bodies and/or small vacuoles. By 20 DPA, most hordein was deposited in the outer 2/3 of the grain's starchy endosperm, with the inner 1/3 being less densely labeled for hordein. In maturing starchy endosperm cells, at 20–30 DPA, the number of protein bodies decreased and the number of small hordein positive vacuoles increased until by 30 DPA few isolated protein bodies could be seen, and all hordein signal was contained in either the central vacuole or small vacuoles. Most hordein‐containing protein bodies had fused with the protein storage vacuoles, and amyloplast‐containing starch granules could be seen silhouetted in front of these central vacuoles. No hordein accumulation was observed in either the aleurone cells or the testa/pericarp (maternal tissue). A similar pattern of many small hordein‐containing vesicles was observed in starchy endosperm and sub‐aleurone cells of developing barley grains at 12–20 DPA (Roustan et al., 2018).

**FIGURE 5:**
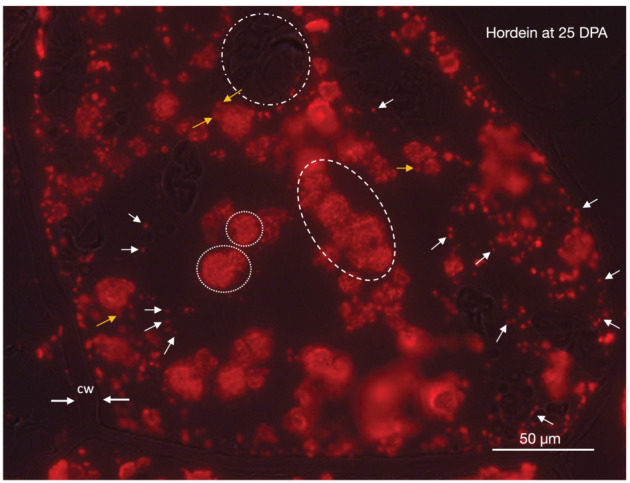
Detail of subcellular location of hordein in a single starchy endosperm cell of barley cv Sloop at 25 days post‐anthesis (DPA) by immunolight microscopy. The plane of section fortuitously allowed exposure of the cellular detail without the interference of amyloplasts. Hordein was seen in protein bodies (*white arrows*) and small vacuoles (*circled with dots*) in the cytoplasm. The small vacuoles had a granular appearance, suggesting that the individual protein bodies have not yet dissipated. Some of the larger protein bodies had begun fusing with larger vacuoles (*orange arrows*). Larger vacuoles appear to have been formed by the fusion of smaller vacuoles (*circled with dashes*). Portions of amyloplasts can be seen in some areas of the micrograph (e.g., *circled with dots and dashes*). The cell wall can be traced around the outside of the cell (*cw*, opposing white arrows). Primary antibody (Ab) was anti‐hordein B4; secondary Ab was anti‐mouse‐Alexa Fluor 568. Scale: 50 μm.

An overview of hordein subcellular location was shown by immunofluorescence microscopy at higher magnification of a single starchy endosperm cell at 25 DPA (Figure 5). Hordein was seen in many different‐sized protein bodies and vacuoles. The contents of large and small vacuoles had a granular appearance due to the presence of individually labeled protein bodies. Larger vacuoles appear to have been formed by the fusion of smaller vacuoles.

**FIGURE 6:**
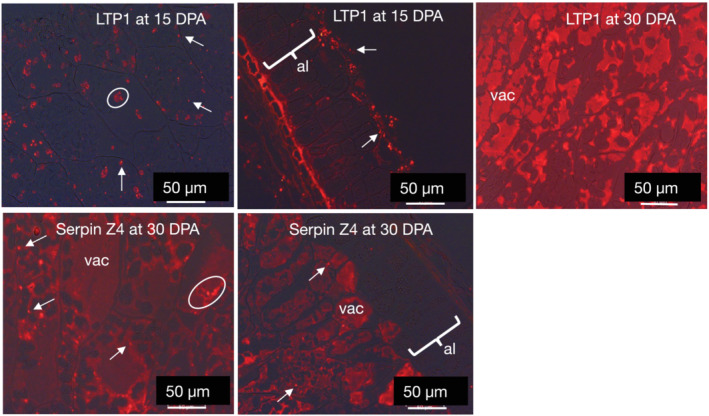
Distribution of lipid transfer protein 1 (LTP1) and serpin Z4 of barley cv Sloop by immunolight microscopy showing accumulating protein bodies (*circled*), aleurone (*al*), and vacuole (*vac*) at the mid (15 days post‐anthesis [DPA]) and late (30 DPA) stages of endosperm development. The position of a single protein body carrying the respective protein label is shown by a white arrow. All scale bars are 50 μm. Primary antibodies (Abs) were rabbit anti‐LTP1 and rabbit anti‐Z4, as appropriate. Secondary Ab was anti‐rabbit‐Alexa Fluor 568.

Immunofluorescence microscopy showed that the subcellular distribution of serpin Z4 and LTP1 was similar to that of hordeins in the starchy endosperm (Figure 6). At 15 DPA, numerous labeled protein bodies were seen, often clumped together due to fusion with small vacuoles and noted as accumulating protein bodies in Figure 6. Serpin Z4 accumulates at 8 DPA and is fully confluent in vacuoles at 15 DPA. LTP1 accumulated in older tissues from 15 DPA onwards. All the results seen for hordein, serpin Z4, and LTP1 agree with the results of western analysis of developing grains (Figure S3). By 30 DPA, most protein bodies had merged with central protein storage vacuoles, which still, however, retain some granularity due to the presence of fragments of protein bodies. Neither LTP1 nor serpin Z4 accumulated in either the aleurone cells or the testa/pericarp (maternal tissue).

**FIGURE 7:**
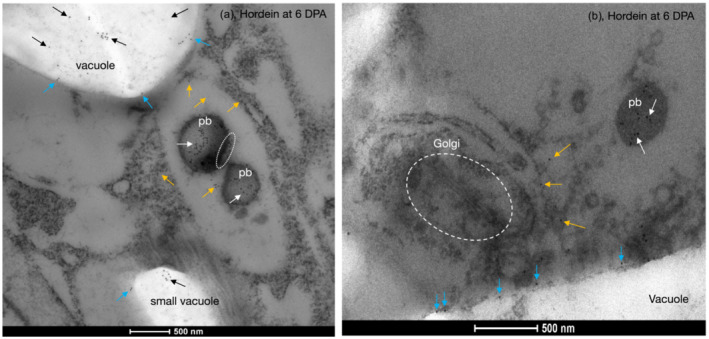
Overall intracellular distribution of hordeins of barley cv Sloop by immunoelectron microscopy. (a) At 6 days post‐anthesis (DPA), gold‐labeled hordeins (white arrows) were inside protein bodies (*pb*), the main vacuole (*vacuole*), or a smaller vacuole (*small vacuole*). Hordeins were also found in the cytoplasm (*orange arrows*) and inside vacuoles as clumps or individually labeled proteins (black arrows). Labeled hordein is also seen outside but is closely aligned with protein bodies (circled with dots). This process is explored in later figures. Individually labeled hordeins were seen close to the vacuole membrane/cytoplasm junction, either still within the cytoplasm or just inside the vacuole membrane (blue arrows). Primary antibody (Ab) was mouse anti‐hordein B4. Secondary Ab was anti‐mouse‐18 nm gold. Scale: 500 nm. (b) At 6 DPA, a Golgi body (*Golgi*, circled) shows no anti‐hordein labeling. Background has been slightly lightened, brightness +20% to enhance the Golgi body. The second Ab‐only controls show one spot per field at this magnification (Figure S12); the arrow designation is the same as for (a), showing additional examples of labeled hordeins close to the cytoplasm/vacuole boundary. All scale bars are 500 nm. Primary anti‐hordein Ab was 23‐3. Secondary Ab was anti‐mouse‐18 nm gold.

Higher resolution of these processes was seen by iEM using a gold‐labeled anti‐mouse secondary antibody to the mouse anti‐hordein antibodies (Figure 7a,b). In a portion of a single cell in the starchy endosperm at 6 DPA, the majority of labeled hordein was seen either inside protein bodies or closely aligned with and just outside protein bodies (Figure 7a,b). It is assumed that these labeled proteins were being imported into the protein bodies. Clumps of hordeins were also present inside the central vacuole, presumably from the merging of protein bodies with the vacuole (Figure 7a). Some individually labeled hordein was also seen close to the vacuole membrane, either just inside or just outside the vacuole membrane (Figure 7a,b). These are presumably due to individual proteins either about to enter or having just entered the vacuole.

**FIGURE 8:**
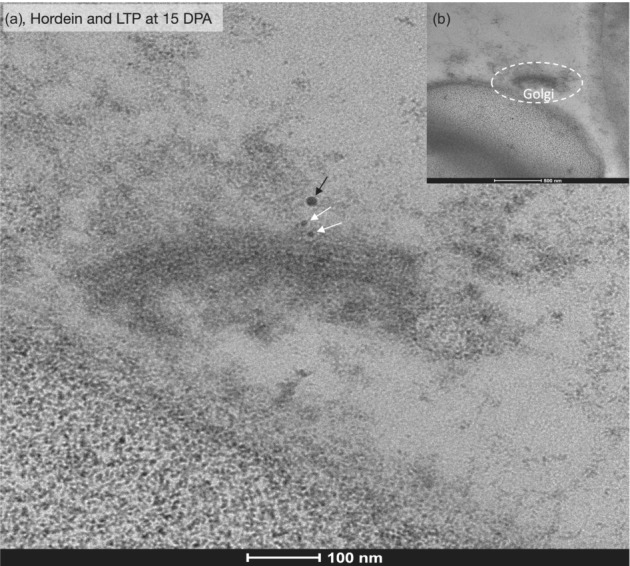
Double labeling of barley cv Sloop showed that anti‐hordein and anti‐lipid transfer protein (LTP) do not label a Golgi body at 15 days post‐anthesis (DPA): (a) Anti‐LTP (black arrow) and anti‐hordein labeling (white arrows) occurred in the cytoplasm but not the Golgi body. Scale bar was 100 nm. (b) Lower magnification of the same area as in (a) shows the Golgi body (*Golgi*, circled with dashes). Scale bar was 500 nm. Primary antibodies (Abs) were anti‐hordein mouse 23‐3 and rabbit anti‐LTP. Secondary Abs were anti‐mouse‐10 nm gold and anti‐rabbit‐18 nm gold.

Single anti‐hordein labeling (Figure 7b) showed that hordein did not label the GA. Double labeling (Figure 8) of a GA showed that neither anti‐hordein nor anti‐LTP labeled the GA.

**FIGURE 9:**
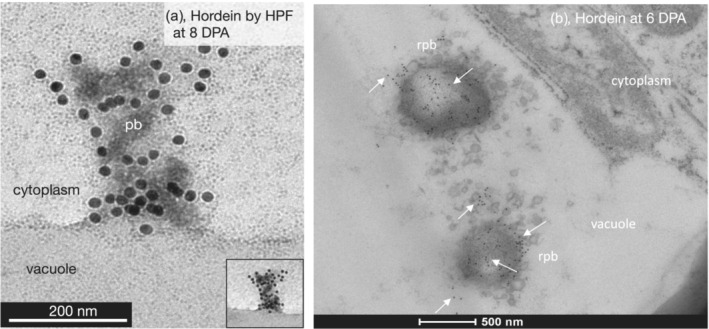
(a) Merging of a protein body with a vacuole of barley cv Sloop visualized by high pressure freezing (HPF)‐immunoelectron microscopy (iEM). At 8 days post‐anthesis (DPA), gold‐labeled hordeins in a protein body (*pb*) enter a vacuole (*vacuole*) from the cytoplasm (*cytoplasm*); scale: 200 nm; the inset shows an image of the same area with lower magnification showing the invagination in the vacuole wall and gold‐labeled hordeins beginning to diffuse into the vacuole. Primary antibody (Ab) was mouse anti‐hordein 23‐3. Secondary Ab was anti‐mouse‐18 nm gold. (b) Decomposition of a protein body inside the vacuole by iEM. At 6 DPA, labeled hordein (*white arrows*) was seen inside and close to remnant protein bodies (*rpb*) inside the vacuole (*vacuole*) and beyond the cytoplasm (*cytoplasm*). The remnant protein body is surrounded by small vesicles, many of which are attached to the bounding membrane of the protein body and are presumably due to decomposition and dissipation of the bounding membrane of the protein body. Primary Ab was mouse anti‐hordein B4. Secondary Ab was anti‐mouse‐18 nm gold. Scale: 500 nm.

Most protein bodies appear intact when they merge with the vacuole. HPF processing showed that the protein body formed an invagination in the vacuole membrane such that the interior of the vacuole was continuous with the interior of the protein body, allowing the labeled protein contents of the protein body to diffuse into the vacuole (Figure 9a). Once inside the vacuole, hordein‐labeled protein bodies begin to dissipate to form remnant protein bodies, which were temporarily surrounded by small vesicles due to the decomposition of the bounding membrane of the protein body (Figure 9b). This dissipation of protein bodies was also seen in Figure 12c. The decomposition of protein bodies within the protein storage vacuole continues until only small fragments of the hordein label remain in the remnant protein body.

**FIGURE 10:**
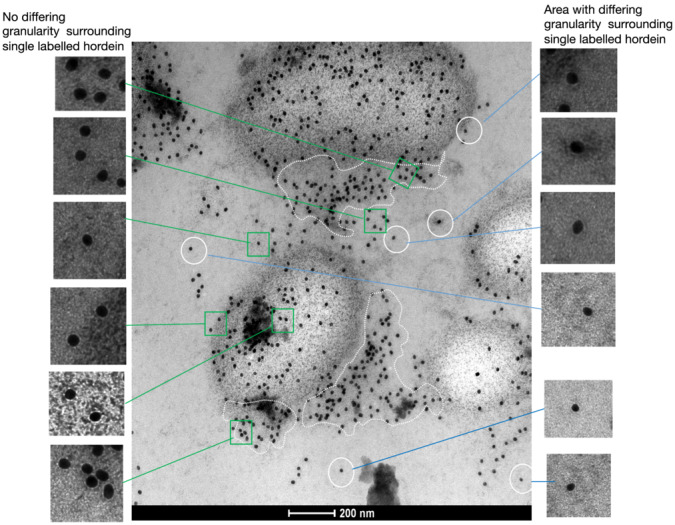
Examination of protein bodies of barley cv Sloop by immunoelectron microscopy following high pressure freezing. At 15 days post‐anthesis, groups of gold‐labeled hordeins in the cytoplasm lay outside the limits of a protein body defined by the granularity of the background and appeared to be imported into the protein body. These proteins lay inside a second area, which was defined by a lower order of granularity (*inside dotted lines*), which was not as dense as the interior of the protein body but denser than the cytoplasm. Some, but not all, individually labeled proteins in the cytoplasm were surrounded by an area differing appearance to the cytoplasm (*circled*) and shown in an expanded view in the insets to the right. The majority of labeled hordeins were not surrounded by this area of differing appearance (green squares) and shown in expanded view in the insets to the left. The contrast of the insets has increased by 93% and the brightness by +33% relative to the original image. Primary antibody (Ab) was mouse anti‐hordein 23‐3. Secondary Ab was anti‐mouse‐18 nm gold. Scale: 100 nm. The original unannotated image is shown in Figure S4. Images showing similar areas of uptake of labeled proteins are shown in Figures 11B,D and S4–S10.

**FIGURE 11:**
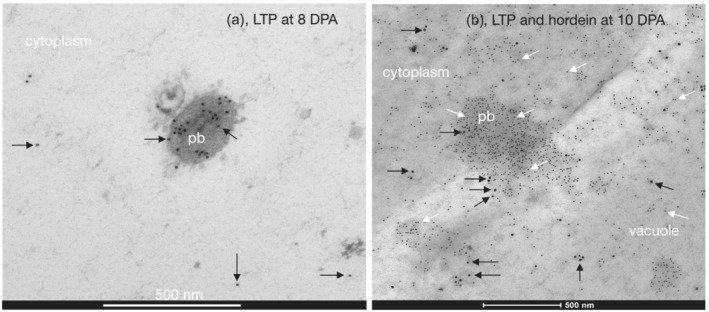
Barley cv Sloop lipid transfer protein (LTP) and hordein colocalize to the same protein body by immunoelectron microscopy. (a) At 8 days post‐anthesis (DPA), 18 nm gold‐labeled LTP (*black arrows*) was seen predominantly inside protein bodies (*pb*) and less frequently in the cytoplasm (*cytoplasm*). Primary antibody (Ab) was rabbit anti‐LTP. Secondary Ab was anti‐rabbit‐18 nm gold. Scale: 500 nm. (b) Double labeling of a protein body (*pb*) entering a vacuole shows that at 10 DPA, 18 nm gold‐labeled LTP (*black arrows*) was seen inside the same protein body that also contained 10 nm gold‐labeled hordein (*white arrows*). Both proteins were also found in the cytoplasm (*cytoplasm*) and vacuole (*vacuole*). Scale: 500 nm. At this DPA, hordein was much more abundant than LTP. Primary Abs were mouse anti‐hordein 23‐3 and rabbit anti‐LTP. Secondary Abs were anti‐rabbit‐18 nm gold and anti‐mouse‐10 nm gold.

**FIGURE 12:**
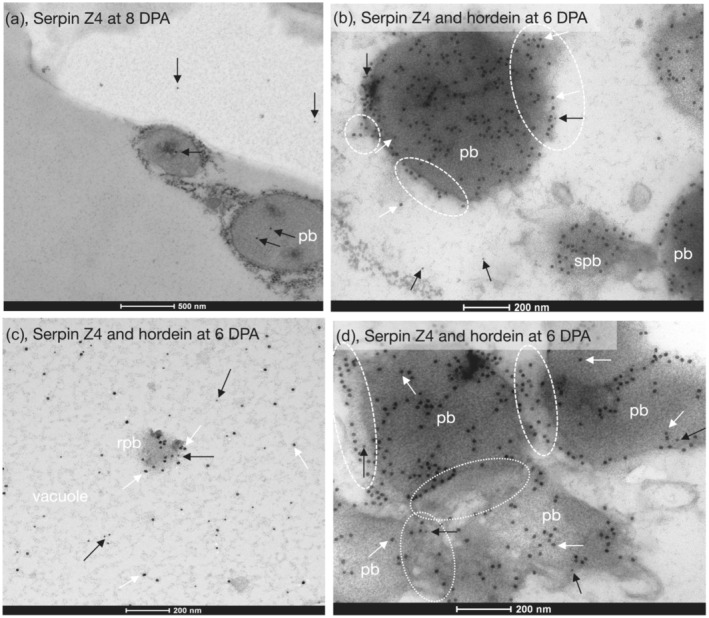
Barley cv Sloop serpin Z4 and hordein colocalize to the same protein body by immunoelectron microscopy. (a) At 8 days post‐anthesis (DPA), 18 nm gold‐labeled serpin Z4 (*black arrows*) was seen inside protein bodies (*pb*), and in the vacuole, primary antibody (Ab) was rabbit anti‐Z4. Secondary Ab was anti‐rabbit‐18 nm gold. Scale: 500 nm. (b) Double labeling showed that at 6 DPA, 10 nm gold‐labeled serpin Z4 (*black arrows*) was seen inside the same protein bodies (*pb*) with 18 nm gold‐labeled hordein (*white arrows*). At this DPA, western blots show that hordein was much more abundant than serpin Z4. Both proteins were also found in the cytoplasm. In the lower right, a small protein body (*spb*) was fusing with a larger protein body (*pb*). Circled areas (*white dashes*) are areas showing labeled proteins merging with the top protein body, similar to those shown in Figure 9. (c) Double labeling showed that at 6 DPA, 18 nm gold‐labeled hordein (*white arrows*) was seen inside the remnant protein body (*rpb*) in the vacuole (*vacuole*) together with 10 nm gold‐labeled serpin Z4 (*black arrows*). Isolated proteins of both serpin Z4 and hordein were also found in the vacuole. (d) Double labeling confirmed that at 6 DPA, 18 nm gold‐labeled hordein (*white arrows*) was seen inside the same protein bodies (*pb*) together with 10 nm gold‐labeled serpin Z4 (*black arrows*). Areas circled (*white dashes*) show labeled proteins merging with the protein bodies similar to those shown in Figure 9. Areas circled (*white dots*) show areas where protein bodies are merging. For (b)–(d), primary Abs were mouse anti‐hordein 23‐3 and rabbit anti‐Z4. Secondary Abs were anti‐mouse‐18 nm gold and anti‐rabbit‐10 nm gold; both scales: 200 nm. These sections were cut from a different block than that used in Figure 10.

Groups of individual gold‐labeled hordeins in the cytoplasm appeared to merge with protein bodies (Figure 10). At 15 DPA, in the cytoplasm of an HPF cell, close examination of a protein body showed that there were two areas associated with the protein body: (i) an area of increased granularity within the circumference traced around the outside of the protein body due to the contents of the protein body and (ii) an area of granularity less dense than the interior of the protein body but denser than the cytoplasm. This area was associated with the protein body but outside the circumference of the protein body. This protein body was in the cytoplasm and not near a vacuole, and it was unlikely that the labeled proteins were being released into this second (“associated”) area from the interior of the protein body. It is also unlikely that this feature is due to protein bodies bursting and releasing hordeins—the outside of the protein bodies appears continuous and does not appear to have ruptured. It was possible to trace a continuous limit around most protein bodies (Figures 10, 11, 12). It is also unlikely that this represents an artifact due to the rapidity of HPF fixation. It is more likely that the rapid HPF process has captured a protein body import mechanism into the protein bodies. Other HPF protein bodies showed similar patterns of aggregation by labeled hordeins (see Figures S4–S10), including sections cut from separate conventionally fixed blocks (Figure 12b,d). By counting the gold particles, which represent individual hordein molecules, approximately 40% of hordein was found outside the protein body in this second (associated) area. When averaged, the proportion in the second (associated) area was remarkably similar. Counting the proportion of hordeins that lay outside the limits of the protein body but within the second (associated) area of increased granularity relative to the cytoplasm for all figures showed that the proportion of hordein in the second area varied from 31.2% to 68.0% (mean ± SE, 43.3 ± 4.5) (Table S2). The agreement between data from different figures may indicate a biological process captured in one instant in the same tissue.

There was a third area containing labeled hordeins, which had a different appearance due to an increased granularity relative to the cytoplasm. In HPF tissue sections, some, but not all, individual hordeins in the cytoplasm were surrounded by an area of different appearance relative to the cytoplasm, suggesting that an increased protein concentration may surround the isolated hordein (Figure 10, right‐hand side [RHS] insets). We do not observe bounding membranes associated with these areas. The increased protein concentration may be due to chaperonins exported from the ER along with the exported hordeins, and they may be involved in maintaining the solubility of the exported hordeins in the cytoplasm. In Figure 10, 12–15 of the 50 gold‐labeled hordeins outside the protein bodies and the associated import areas discussed above had this denser surrounding granularity. Labeled proteins inside either vacuoles or protein bodies did not have this appearance (Figure 10, left‐hand side [LHS] insets). These areas of increased granularity were not apparent in conventionally fixed tissues, presumably because the structures were destroyed during fixation. It is likely that these structures were previously undetected but form part of the suite of plant vesicles recently documented (van de Meene et al., 2017). We suggest that the presence/absence of the surrounding granularity may represent two pathways by which hordeins reach the cytoplasm. One pathway is directly via exocytosis from the ER lumen (with surrounding chaperonins/proteins from the ER), and the second pathway is involving hordeins that have lost their surrounding structure. These encapsulated hordeins appear to lose their encapsulation after absorption into protein bodies. Un‐encapsulated proteins may then diffuse from within the protein body and either re‐enter other protein bodies or vacuoles or remain within the cytoplasm.

Gold‐labeled LTP was also seen inside protein bodies (Figure 11a). Double labeling showed that gold‐labeled LTP was seen inside the same protein bodies that contained gold‐labeled hordein. Both proteins were also found in the cytoplasm and vacuole (Figure 11b).

Gold‐labeled serpin Z4 was also seen inside protein bodies and in the vacuole (Figure 12a). Double labeling showed that gold‐labeled serpin Z4 also occurred inside the same protein bodies that contained gold‐labeled hordein (Figure 12b,d). In Figure 12b, a small protein body was also seen fusing with a larger protein body. Both labeled proteins also co‐occurred in the same remnant protein body within the vacuole and were also found isolated within the vacuole (Figure 12c). At this DPA, western blots show that serpin Z4 is not a dominant protein (Figure S3A). In this figure, areas of individual labeled hordeins occurred closely associated with but outside the limits of the protein bodies (Figure 12b,d), similar to those seen in Figure 10. The sections for Figure 10 were cut from a different sample than that used in Figure 12.

## 4 DISCUSSION AND CONCLUSION

### 4.1 Hordein, serpin Z4, and LTP1 accumulate in the starchy endosperm

Neither hordein, serpin Z4, nor LTP accumulated to any significant degree in either the aleurone or testa/pericarp and were largely confined to the starchy endosperm. In barley, LTP1 was cloned from barley aleurone strips (Mundy & Rogers, 1986). However, here, we show that in developing cv Sloop grains, LTP1 accumulated predominantly in starchy endosperm. Serpin Z4 was previously localized to the central and peripheral starchy endosperm, sub‐aleurone, and at lower levels, to the aleurone (Roberts et al., 2003). Here, we show that hordein, serpin Z4, and LTP1 accumulate predominantly in the starchy endosperm of developing cv Sloop barley grains.

### 4.2 The appearance and uptake of isolated hordeins

**FIGURE 13:**
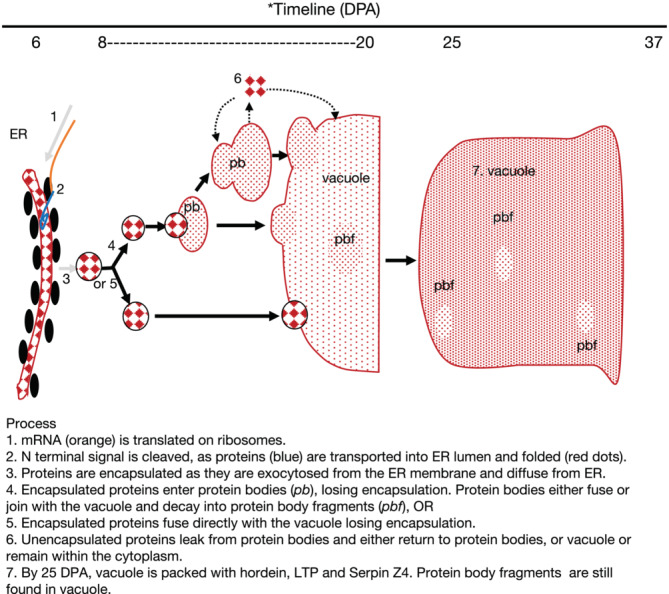
Schematic representation for the synthesis, transport, and accumulation of storage proteins in the starchy endosperm during barley grain development. These processes occurred continuously post‐endosperm cellularization throughout grain development but were more common during the time range indicated as follows: (i) Day 6: The hordein N‐terminal signal peptide is post‐translationally cleaved as peptides accumulated in the endoplasmic reticulum (ER) lumen and protein bodies (*pb*). (ii) Days 8–20: Hordein is exocytosed from ER and encapsulated by ER‐derived proteins. Exported hordeins fuse with either protein bodies (*pb*) or vacuole (*vacuole*). Most hordein is in protein bodies by Day 15, as are lipid transfer protein (LTP) and serpin Z4. Protein bodies began to fuse and dissipate within vacuoles. (iii) Day 25: The majority of hordein, LTP, and serpin Z4 are in the central vacuoles, which have expanded to almost fill the cell; some protein body fragments are still visible in the vacuoles. (iv) Day 37: Desiccation is complete. The testa/pericarp have collapsed. Protein body fragments (*pbf*) were still visible in vacuoles. Golgi apparatus, cell wall, nucleus, mitochondria, and amyloplasts were unlabeled by hordein, LTP1, or serpin Z4. Steps not shown by our data are shown by gray arrows. DPA, days post‐anthesis.

Biochemical evidence has long been available that hordeins are synthesized and temporarily accumulate in the ER. The hordeins are exocytosed from the ER lumen to the cytoplasm (schematically summarized in Figure 13) (Cameron‐Mills et al., 1978; van de Meene et al., 2017). Some cytoplasmic hordeins appeared to be surrounded by an area that appeared different from the surrounding cytoplasm, possibly due to encapsulation by abundant proteins such as chaperonins, which may be required to keep these hydrophobic proteins soluble in the aqueous environment of the cytoplasm and which may have been exocytosed from the ER along with hordein. However, we have no evidence for encapsulation of either serpin Z4 or LTP1, although we assume that they follow the same path as hordeins. The export of wheat gliadin from the ER to protein bodies in encapsulated vesicles, which are internalized into vacuoles by autophagy, has been proposed for wheat (Galili et al., 1993).

The cytoplasmic hordeins appeared to be absorbed by protein bodies. High concentrations of gold‐labeled hordeins in the cytoplasm lay close to protein bodies, in aggregates, just outside the limits of the protein bodies defined by the granularity inside the protein body. These proteins lay inside a second area, which was defined by a lower order of granularity compared to the interior of the protein body but was also distinguishable from the cytoplasm. It is likely this represents an uptake mechanism caught by the HPF method.

It appears that a proportion of the hordein within protein bodies may escape and then either re‐enter protein bodies or vacuoles or remain within the cytoplasm. Protein bodies eventually fuse with the central vacuole and begin to decompose. Yeast carboxypeptidase moves from the rough endoplasmic reticulum (RER) to the vacuole with a half‐life of 5 min (Winther et al., 1991). ER exit sites in *Nicotiana tabacum* BY‐2 cells are stationary but extremely short‐lived, with a half‐life of <10 sec (Yang et al., 2005). It is likely that individual steps in hordein transport also occur rapidly. However, examples of each stage can be found in sections isolated from a range of DPA, showing that the processes occurred continuously during starchy endosperm development following cellularization in barley grains. The overall effect is a net transport of hordeins from the site of synthesis on the ER into the protein storage vacuoles. These processes also occur continuously throughout grain development in wheat (Moore et al., 2016).

### 4.3 Glycosylation of hordeins

The situation regarding hordein glycosylation remains unresolved. Our iEM clearly shows that hordeins (and LTP) are not labeled in the GA (Figures 7b and 12).

Our tandem mass spectrometry (MS) data from purified hordeins were searched for evidence of glycosylated hordeins based on modifications commonly observed, for example, glycan residue mass of 162 (hexose sugar), 203 (HexNAc sugar), or 365 (Hex + HexNAc). The data showed no evidence that any of the hordeins were glycosylated. However, the sample preparation approach and data acquisition were not specifically generated for the purpose of glycosylation analysis, and our negative MS evidence may not be proof of absence (Colgrave, personal communication, March 4, 2024) (Tanner et al., 2019).

In contrast, Snegaroff et al. (2013) presented evidence for glycosylation of at least a proportion of the minor hordein, γ3‐hordein. MS–MS showed that a proportion of γ3‐hor was N‐glycosylated at an atypical Asn_140_‐Leu‐Cys_142_ site in a γ3‐hor purified protein band. N‐linked glycosylation usually occurs at Asn‐X‐Ser/Thr, where X is any amino acid other than Pro or Asp (Jones et al., 2005). MS–MS evidence from γ3‐hor cyanogen bromide/trypsin/Glu‐C fragments showed a parent ion at m/z = 1899 corresponding to the peptide Q_136_–E_144_ containing the proposed glycosylation site at N_140_. Fragments of this parent ion produced the mass signature of two N‐acetylhexosamines and two hexoses, which confirms that the γ3‐hor protein was initially glycosylated in the ER (Jones et al., 2005). Significantly, they also observe pentose–hexose residues from the parent ion, confirming that an additional pentose residue was then added by glycosylation in the GA (Strasser et al., 2021). This indicates that at least some γ3‐hor proteins were trafficked through the GA.

It may be that although the antibody used here was capable of identifying all hordeins, including γ‐1,‐2,‐3‐hor, on a western blot, further experimental work is required to resolve the location of γ‐1‐hor.

### 4.4 Hordein, serpin Z4, and LTP1 co‐populate the same protein bodies

We also examined the location of serpin Z4 and LTP1. Double labeling unexpectedly showed that these three proteins—hordein, serpin Z4, and LTP1—shared the same protein bodies. The pathway outlined in Figure 13 seems to be common for hordein, LTP1, and serpin Z4 in developing barley grains. Serpin Z4 is synthesized without a signal peptide, unlike the hordeins (Brandt et al., 1990), so it is puzzling how it is directed to the ER and then protein bodies. We provide iEM evidence that serpin Z4 is localized within protein bodies, the cytoplasm, and the storage vacuole in barley, but we have no evidence of how serpin Z4 proceeds from the site of synthesis to accumulate in protein bodies and vacuoles. An internal hydrophobic region of 21 amino acids (residues 36–56) may serve as a signal for targeting the serpin Z4 polypeptide into the lumen of the ER (Brandt et al., 1990). From the ER lumen, serpin Z4 may be directed to protein bodies and hence to the central storage vacuole in the same manner as hordeins and LTP1.

### 4.5 Hordeins and LTP1 do not transit the GA

Double labeling showed that anti‐LTP1 and anti‐hordein labeling occurred in the protein bodies and cytoplasm but not the GA. It would be expected to label the vesicles at the termini of the Golgi cisternae, as seen in wheat (Kim et al., 1988). It is likely that in barley, the water‐insoluble hordeins and the soluble albumins and globulins follow different subcellular trafficking routes. Whereas the glycosylated albumins and globulins would typically pass through the GA (Shewry & Halford, 2002), we show here that at least the dominant B‐ and C‐hordeins and LTP1 are unlikely to transit the GA.

### 4.6 Further research

It would be interesting to examine the biochemical nature of proteins that closely associate with hordeins in protein bodies, isolated hordeins within the cytoplasm, and hordeins within the ER (isolated microsomes). Biochemical isolation of these fractions derived from extracts of developing barley grains would be difficult but possible. Immuno‐pull‐down experiments with anti‐hordein antibodies could reveal the nature of proteins that closely associate with hordeins in these three compartments.

It would also be interesting to examine live images of the hordein transport process from beginning to end by laser confocal and spinning disk microscopy using heterologously tagged hordeins. This would potentially reveal much more detail than is seen by examining fixed sections. However, it is very difficult to transform barley; only one cultivar, Golden Promise, is transformable (Harwood, 2011), and transforming that cv with suitably tagged proteins to illuminate the multiple compartments containing hordein, ER, GA, and storage vesicles that may be examined by confocal microscopy involves a large body of work over several years and is well beyond the scope of this manuscript.

## AUTHOR CONTRIBUTIONS

**Gregory Tanner:** Conceptualization; methodology; investigation; writing—review and editing. **Allison van de Meene:** Conceptualization; methodology; investigation; writing—review and editing. **Anthony Bacic:** resources; writing—review and editing; funding acquisition.

## CONFLICT OF INTEREST STATEMENT

No conflict of interest was declared.

## Supporting information


**Figure S1.** Pileup analysis of hordeins to identify 8 family specific peptides (P1‐P8, coloured above the sequences). Repetitive sequences in the D‐hordein are underlined. Peptides 1 (B‐hor), 3 (B‐hor), 4 (B‐hor), 5 (C‐hor) did not produce MAbs which reacted to western blots. P2 gave rise to the general anti‐hordein antibody B4, used here. The ~20 amino‐acid N‐terminus indicated thus, is removed during post translational transit into the ER lumen.
**Table S1.** Peptides used to raise 1st and 2nd round anti‐hordein monoclonals.
**Figure S2.** Reproduced with permission from Figure 2 (Tanner et al., 2019). Hordein Accumulation in Developing Barley Grains. Frontiers in Plant Science, DOI: 10.3389/fpls.2019.00649. A. Fresh weight of developing barley grains and B. Hordein content by ELISA, calibrated against total Sloop hordein (mg/gFWt●, left y‐axis), or g/100 g protein (■, right y‐axis). Means (n = 4) ± SE are shown; no error bars are shown when SE < symbol size.
**Figure S3.** Reproduced with permission from Figure 3, Tanner et al “Hordein Accumulation in Developing Barley Grains”. Frontiers in Plant Science, DOI: 10.3389/fpls.2019.00649. A, mid‐stage accumulation of Serpin Z4: (*a*), serpin Z7 (47.3 kDA), (*b*), serpin Z4 (44.3 kDa), (*c*) serpin Z4 minus 4 kDa active loop (41.6 kDa). 1Ab rabbit anti‐Z4 V6175; 2Ab Amersham (GE Heath Aust) antirabbit‐HRP. B, late‐stage accumulation mature LTP (9.0 kDa) (*f*). 1Ab rabbit anti‐LTP V6177, 2Ab Amersham (GE Health Australia) antirabbit‐HRP. C, early‐stage accumulation of D‐hordein (93.9 kDa) (*g*), C‐hordeins (70.5, 63.7 and 55.6 kDa) (*h, i, j*), B‐hor (47.8 kDa) (*k*), partly obscuring γ‐1‐hordein (45.0 kDA) (*l*), γ‐2‐hor (40.0 kDa) (*m*), γ‐3‐hordein (38.0 kDa) (*n*). 1Ab Sigma anti‐gluten HRP (the same pattern was observed for mouse anti‐hordeins B4, and 23‐3 with 2 Ab Amersham (GE Health Australia) anti‐mouse‐HRP).
**Figure S4.** The original unannotated image that is shown in Figure 10. For clarity all annotations have been removed.
**Figure S5.** Another example of hordeins outside a protein body, same section as Figure 10.
**Figure S6.** Another example from the same section as Figure 10.
**Figure S7.** Another example of hordeins outside PB from a different section, same block to Figure 10.
**Figure S8.** Another example of hordeins outside PB from a different section, same block to Figure 10.
**Figure S9.** Another example of hordeins outside PB, from a different section, same block to Figure 10.
**Figure S10.** Another example of hordeins outside PB, from a different section, same block as Figure 10.
**Table S2.** Proportion of labelled hordeins outside the limits of the protein body.
**Figure S11.** Second Ab alone control of barley cv Sloop, with Invitrogen donkey anti‐mouse‐IgG‐Alexa Flour 568 at (A) 8 DPA, scale bar 50 μm; (B) 30 DPA, scale bar 100 μm. Some slight autofluorescence occurs at the testa/pericarp, at 5 oclock. Goat anti‐rabbit IgG‐Alexa Fluor 568 (Abcam) alone, also did not generate a signal but the original image was lost.
**Figure S12.** Second Ab control of barley cv Sloop, at 15 DPA with AR‐10 nm gold (A) showing protein body; (B) showing cytoplasm, scale bars 500 nm. AR‐10 nm gold shows one particle per field at this magnification (*white arrow*).
**Figure S13.** Second Ab control of barley cv Sloop with AR‐18 nm gold at 8 DPA (A) showing unlabeled areas of cytoplasm (*cytoplasm*) and vacuole (*vacuole*); (B) unlabeled protein body (*pb*) and cytoplasm (*cytoplasm*) . Scale bars are 200 nm.
**Figure S14.** Second Ab control of barley cv Sloop at 10 DPA, with AM‐10 nm gold (A) showing 1 particle (*white arrow*) in an area of cytoplasm (*cytoplasm*). The dark area at six o'clock is a fold in the section; (B) showing unlabeled cell wall (*cell wall*) and areas of cytoplasm (*cytoplasm*). Scale bars 500 nm.
**Figure S15.** Second Ab control of barley cv Sloop at 15 DPA, with AM‐18 nm gold (A, B) each showing one particle per field (white arrows) in an area of cytoplasm (*cytoplasm*). Scale bars are 500 nm (A) and 200 nm (B).

**Data S1.** Peer Review.

## Data Availability

All data are supplied in this manuscript or in the supporting information.

## References

[pld3591-bib-0001] Anderson, O. D. (2013). The B‐hordein prolamin family of barley. Genome, 56, 179–185. 10.1139/gen-2013-0016 23659702

[pld3591-bib-0002] Arcalis, E. , Ibl, V. , Hilscher, J. , Rademacher, T. , Avesani, L. , Morandini, F. , Bortesi, L. , Pezzotti, M. , Vitale, A. , Pum, D. , De Meyer, T. , Depicker, A. , & Stoger, E. (2019). Russell‐like bodies in plant seeds share common features with prolamin bodies and occur upon recombinant protein production. Frontiers in Plant Science, 10, 777. 10.3389/fpls.2019.00777 31316529 PMC6611407

[pld3591-bib-0003] Bethke, P. C. , & Jones, R. L. (2000). Vacuoles and prevacuolar compartments. Current Opinion in Plant Biology, 3, 469–475. 10.1016/S1369-5266(00)00115-1 11074377

[pld3591-bib-0004] Brandt, A. (1976). Endosperm protein formation during kernel development of wild type and a high‐lysine barley mutant. Cereal Chemistry, 53, 890–901.

[pld3591-bib-0005] Brandt, A. , Svendsen, I. , & Hejgaard, J. (1990). A plant serpin gene—Structure, organization and expression of the gene encoding barley protein‐Z4. European Journal of Biochemistry, 194, 499–505. 10.1111/j.1432-1033.1990.tb15644.x 2269280

[pld3591-bib-0006] Breu, V. , Guerbette, F. , Kader, J.‐C. , Kannangara, C. G. , Svensson, B. , & Von Wettstein‐Knowles, P. (1989). A 10 kD barley basic protein transfers phosphatidylcholine from liposomes to mitochondria. Carlsberg Research Communications, 54, 81–84. 10.1007/BF02907587

[pld3591-bib-0007] Cameron‐Mills, V. , & Brandt, A. (1988). A gamma‐hordein gene. Plant Molecular Biology, 11, 449–461. 10.1007/BF00039026 24272402

[pld3591-bib-0008] Cameron‐Mills, V. , Ingversen, J. , & Brandt, A. (1978). Transfer of in vitro synthesized barley endosperm proteins into the lumen of the endoplasmic reticulum. Carlsberg Research Communications, 4, 91–102.

[pld3591-bib-0009] Cameron‐Mills, V. , & Madrid, S. M. (1989). The signal peptide cleavage site of a B1 hordein determined by radiosequencing of the in vitro synthesized and processed polypeptide. Carlsberg Research Communications, 54, 181–192. 10.1007/BF02904472

[pld3591-bib-0010] Cohen, M. , & Fluhr, R. (2018). Noncanonical interactions between serpin and β‐amylase in barley grain improve β‐amylase activity in vitro. Plant Direct, 2, e00054. 10.1002/pld3.54 31245723 PMC6508567

[pld3591-bib-0011] Cui, Y. , Zhao, Q. , Hu, S. , & Jiang, L. (2020). Vacuole biogenesis in plants: How many vacuoles, how many models? Trends in Plant Science, 25, 538–548. 10.1016/j.tplants.2020.01.008 32407694

[pld3591-bib-0012] Ebert, B. , Rautengarten, C. , McFarlane, H. E. , Rupasinghe, T. , Zeng, W. , Ford, K. , Scheller, H. V. , Bacic, A. , Roessner, U. , Persson, S. , & Heazlewood, J. L. (2018). A Golgi UDP‐GlcNAc transporter delivers substrates for N‐linked glycans and sphingolipids. Nature Plants, 4, 792–801. 10.1038/s41477-018-0235-5 30224661

[pld3591-bib-0013] Edqvist, J. , Blomqvist, K. , Nieuwland, J. , & Salminen, T. A. (2018). Plant lipid transfer proteins: Are we finally closing in on the roles of these enigmatic proteins? Journal of Lipid Research, 59, 1374–1382. 10.1194/jlr.R083139 29555656 PMC6071764

[pld3591-bib-0014] Entwistle, J. (1988). Primary structure of a C‐hordein gene from barley. Carlsberg Research Communications, 53, 247–258. 10.1007/BF02907181 3255313

[pld3591-bib-0015] Ferreira, M. M. , Santos, A. S. , Santos, A. S. , Zugaib, M. , & Pirovani, C. P. (2023). Plant serpins: Potential inhibitors of serine and cysteine proteases with multiple functions. Plants, 12, 3619. 10.3390/plants12203619 37896082 PMC10609998

[pld3591-bib-0016] Galili, G. , Altschuler, Y. , & Levanony, H. (1993). Assembly and transport of seed storage proteins. Trends in Cell Biology, 3, 437–442. 10.1016/0962-8924(93)90033-W 14731890

[pld3591-bib-0017] Gu, Y. Q. , Anderson, O. D. , Londeore, C. F. , Kong, X. Y. , Chibbar, R. N. , & Lazo, G. R. (2003). Structural organization of the barley D‐hordein locus in comparison with its orthologous regions of wheat genomes. Genome, 46, 1084–1097. 10.1139/g03-071 14663527

[pld3591-bib-0018] Harwood, W. A. (2011). Advances and remaining challenges in the transformation of barley and wheat. Journal of Experimental Botany, 63, 1791–1798. 22140237 10.1093/jxb/err380

[pld3591-bib-0019] Hilscher, J. , Kapusi, E. , Stoger, E. , & Ibl, V. (2016). Cell layer‐specific distribution of transiently expressed barley ESCRT‐III component HvVPS60 in developing barley endosperm. Protoplasma, 253, 137–153. 10.1007/s00709-015-0798-1 25796522 PMC4712231

[pld3591-bib-0020] Ibl, V. (2019). ESCRTing in cereals: Still a long way to go. Science China Life Sciences, 62, 1144–1152. 10.1007/s11427-019-9572-9 31327097

[pld3591-bib-0021] Ibl, V. , Kapusi, E. , Arcalis, E. , Kawagoe, Y. , & Stoger, E. (2014). Fusion, rupture, and degeneration: The fate of in vivo‐labelled PSVs in developing barley endosperm. Journal of Experimental Botany, 65, 3249–3261. 10.1093/jxb/eru175 24803499 PMC4071841

[pld3591-bib-0022] Jones, J. , Krag, S. S. , & Betenbaugh, M. J. (2005). Controlling N‐linked glycan site occupancy. Biochimica et Biophysica Acta (BBA) ‐ General Subjects, 1726, 121–137. 10.1016/j.bbagen.2005.07.003 16126345

[pld3591-bib-0023] Kim, W. T. , Franceschi, V. R. , Krishnan, H. B. , & Okita, T. W. (1988). Formation of wheat protein bodies: Involvement of the Golgi apparatus in gliadin transport. Planta, 176, 173–182. 10.1007/BF00392442 24220770

[pld3591-bib-0024] Kreis, M. , Rahman, S. , Forde, B. G. , Pywell, J. , Shewry, P. R. , & Miflin, B. J. (1983). Sub‐families of hordein mRNA encoded at the Hor2 locus of barley. Molecular General Genetics, 191, 194–200. 10.1007/BF00334813

[pld3591-bib-0055] Kreis, M. , & Shewry, P. R. (1992). The control of protein synthesis in developing barley seeds. In P. R. Shewry Barley: Genetics, biochemistry, molecular biology and biotechnology (pp. 319–333). CAB International.

[pld3591-bib-0025] Levanony, H. , Rubin, R. , Altschuler, Y. , & Galili, G. (1992). Evidence for a novel route of wheat storage proteins to vacuoles. Journal of Cell Biology, 119, 1117–1128. 10.1083/jcb.119.5.1117 1447291 PMC2289714

[pld3591-bib-0026] Miflin, B. J. , Burgess, S. R. , & Shewry, P. R. (1981). The development of protein bodies in the storage tissues of seeds: Subcellular separations of homogenates of barley, maize, and wheat endosperms and of pea cotyledons. Journal of Experimental Botany, 32, 199–219. 10.1093/jxb/32.1.199

[pld3591-bib-0027] Møgelsvang, S. , & Simpson, D. J. (1998). Changes in the levels of seven proteins involved in polypeptide folding and transport during endosperm development of two barley genotypes differing in storage protein localisation. Plant Molecular Biology, 36, 541–552. 10.1023/A:1005916427024 9484449

[pld3591-bib-0028] Moore, K. L. , Tosi, P. , Palmer, R. , Hawkesford, M. J. , Grovenor, C. R. M. , & Shewry, P. R. (2016). The dynamics of protein body formation in developing wheat grain. Plant Biotechnology Journal, 14, 1876–1882. 10.1111/pbi.12549 26898533 PMC4988504

[pld3591-bib-0029] Mundy, J. , & Rogers, J. C. (1986). Selective expression of a probable amylase/protease inhibitor in barley aleurone cells: Comparison to the barley amylase/subtilisin inhibitor. Planta, 169, 51–63. 10.1007/BF01369775 24232429

[pld3591-bib-0030] Rechinger, K. B. , Bougri, O. V. , & Cameron‐Mills, V. (1993). Evolutionary relationship of the members of the sulphur‐rich hordein family revealed by common antigenic determinants. Theoretical and Applied Genetics, 85, 829–840. 10.1007/BF00225026 24196057

[pld3591-bib-0031] Rechinger, K. B. , Simpson, D. J. , Svendsen, I. , & Cameron‐Mills, V. (1993). A role for gamma 3 hordein in the transport and targeting of prolamin polypeptides to the vacuole of developing barley endosperm. The Plant Journal, 4, 841–853. 10.1046/j.1365-313X.1993.04050841.x 7506098

[pld3591-bib-0032] Roberts, T. H. , Marttila, S. , Rasmussen, S. K. , & Hejgaard, J. (2003). Differential gene expression for suicide‐substrate serine proteinase inhibitors (serpins) in vegetative and grain tissues of barley. Journal of Experimental Botany, 54, 2251–2263. 10.1093/jxb/erg248 14504298

[pld3591-bib-0033] Roustan, V. , Hilscher, J. , Weidinger, M. , Reipert, S. , Shabrangy, A. , Gebert, C. , Dietrich, B. , Dermendjiev, G. , Schnurer, M. , Roustan, P.‐J. , Stoger, E. , & Ibl, V. (2020). Protein sorting into protein bodies during barley endosperm development is putatively regulated by cytoskeleton members, MVBs and the HvSNF7s. Scientific Reports, 10, 1864. 10.1038/s41598-020-58740-x 32024857 PMC7002727

[pld3591-bib-0034] Roustan, V. , Roustan, P.‐J. , Weidinger, M. , Reipert, S. , Kapusi, E. , Shabrangy, A. , Stoger, E. , Weckwerth, W. , & Ibl, V. (2018). Microscopic and proteomic analysis of dissected developing barley endosperm layers reveals the starchy endosperm as prominent storage tissue for ER‐derived hordeins alongside the accumulation of barley protein disulfide isomerase (HvPDIL1‐1). Frontiers in Plant Science, 9, 1248. 10.3389/fpls.2018.01248 30250475 PMC6139375

[pld3591-bib-0035] Shewry, P. R. , Bunce, N. A. C. , Kreis, M. , & Forde, B. G. (1985). Polymorphism at the Hor‐1 locus of barley (*Hordeum vulgare* L.). Biochemical Genetics, 23, 391–405. 10.1007/BF00499082 2994625

[pld3591-bib-0036] Shewry, P. R. , & Halford, N. G. (2002). Cereal seed storage proteins: Structures, properties and role in grain utilization. Journal of Experimental Botany, 53, 947–958. 10.1093/jexbot/53.370.947 11912237

[pld3591-bib-0037] Shewry, P. R. , Kreis, M. , Parmar, S. , Lew, E. J. L. , & Kasarda, D. D. (1985). Identification of gamma‐type hordeins in barley. FEBS Letters, 190, 61–64. 10.1016/0014-5793(85)80427-0

[pld3591-bib-0038] Shimada, T. , Takagi, J. , Ichino, T. , Shirakawa, M. , & Hara‐Nishimura, I. (2018). Plant vacuoles. Annual Review of Plant Biology, 69, 123–145. 10.1146/annurev-arplant-042817-040508 29561663

[pld3591-bib-0039] Skriver, K. , Leah, R. , Müller‐Uri, F. , Olsen, F. L. , & Mundy, J. (1992). Structure and expression of the barley lipid transfer protein gene Ltp1. Plant Molecular Biology, 18, 585–589. 10.1007/BF00040674 1536930

[pld3591-bib-0040] Snegaroff, J. , Bouchez‐Mahiout, I. , Smaali, M. , Pecquet, C. , Raison‐Peyron, N. , Jolivet, P. , & Laurière, M. (2013). Barley gamma 3‐hordein: Glycosylation at an atypical site, disulfide bridge analysis, and reactivity with IgE from patients allergic to wheat. Biochimica et Biophysica Acta Proteins and Proteomics, 1834, 395–403. 10.1016/j.bbapap.2012.07.016 22885023

[pld3591-bib-0041] Sorensen, M. B. , Cameron‐Mills, V. , & Brandt, A. (1989). Transcriptional and post‐transcriptional regulation of gene expression in developing barley endosperm. Molecular and General Genetics, 217, 195–201. 10.1007/BF02464881

[pld3591-bib-0042] Strasser, R. , Seifert, G. , Doblin, M. S. , Johnson, K. L. , Ruprecht, C. , Pfrengle, F. , Bacic, A. , & Estevez, J. M. (2021). Cracking the “sugar code”: A snapshot of N‐ and O‐glycosylation pathways and functions in plants cells. Frontiers in Plant Science, 12, 640919. 10.3389/fpls.2021.640919 33679857 PMC7933510

[pld3591-bib-0043] Tan, X. , Li, K. , Wang, Z. , Zhu, K. , Tan, X. , & Cao, J. (2019). A review of plant vacuoles: Formation, located proteins, and functions. Plants (Basel), 8(9), 327. 10.3390/plants8090327 31491897 PMC6783984

[pld3591-bib-0054] Tanner, G. J. , Blundell, M. J. , Colgrave, M. L. , Howitt, C. A. (2013). Quantification of Hordeins by ELISA: The Correct Standard Makes a Magnitude of Difference. Plos One, 8, e56456. 10.1371/journal.pone.0056456 23509607 PMC3585327

[pld3591-bib-0044] Tanner, G. J. , Blundell, M. J. , Colgrave, M. L. , & Howitt, C. A. (2016). Creation of the first ultra‐low gluten barley (*Hordeum vulgare* L.) for coeliac and gluten‐intolerant populations. Plant Biotechnology Journal, 14, 1139–1150. 10.1111/pbi.12482 26427614 PMC5054857

[pld3591-bib-0045] Tanner, G. J. , Colgrave, M. L. , Blundell, M. J. , Howitt, C. A. , & Bacic, A. (2019). Hordein accumulation in developing barley grains. Frontiers in Plant Science, 10, 649. 10.3389/fpls.2019.00649 31156692 PMC6532529

[pld3591-bib-0046] Tanner, G. J. , Colgrave, M. L. , & Howitt, C. A. (2014). Gluten, celiac disease, and gluten intolerance and the impact of gluten minimization treatments with prolylendopeptidase on the measurement of gluten in beer. Journal of the American Society of Brewing Chemists, 72, 36–50.

[pld3591-bib-0047] Tosi, P. , Parker, M. , Gritsch, C. S. , Carzaniga, R. , Martin, B. , & Shewry, P. R. (2009). Trafficking of storage proteins in developing grain of wheat. Journal of Experimental Botany, 60, 979–991. 10.1093/jxb/ern346 19174462 PMC2652050

[pld3591-bib-0048] Ullrich, S. E. , Rasmussen, U. , Høyer‐Hansen, G. , & Brandt, A. (1986). Monoclonal antibodies to hordein polypeptides. Carlsberg Research Communications, 51, 381–399. 10.1007/BF02907314

[pld3591-bib-0049] van de Meene, A. M. , Doblin, M. S. , & Bacic, A. (2017). The plant secretory pathway seen through the lens of the cell wall. Protoplasma, 254, 75–94. 10.1007/s00709-016-0952-4 26993347

[pld3591-bib-0050] Wilson, S. M. , & Bacic, A. (2012). Preparation of plant cells for transmission electron microscopy to optimise immunogold labelling of carbohydrate and protein epitopes. Nature Protocols, 7, 1716–1727. 10.1038/nprot.2012.096 22918389

[pld3591-bib-0051] Wilson, S. M. , Burton, R. A. , Doblin, M. S. , Stone, B. A. , Newbigin, E. J. , Fincher, G. B. , & Bacic, A. (2006). Temporal and spatial appearance of wall polysaccharides during cellularization of barley (*Hordeum vulgare*) endosperm. Planta, 224, 655–667. 10.1007/s00425-006-0244-x 16532317

[pld3591-bib-0052] Winther, J. R. , Stevens, T. H. , & Kielland‐Brandt, M. C. (1991). Yeast carboxypeptidase Y requires glycosylation for efficient intracellular transport, but not for vacuolar sorting, in vivo stability, or activity. European Journal of Biochemistry, 197, 681–689. 10.1111/j.1432-1033.1991.tb15959.x 2029899

[pld3591-bib-0053] Yang, Y. , Elamawi, R. , Bubeck, J. , Pepperkok, R. , Ritzenthaler, C. , & Robinson, D. (2005). Dynamics of COPII vesicles and the Golgi apparatus in cultured *Nicotiana tabacum* BY‐2 cells provides evidence for transient association of Golgi stacks with endoplasmic reticulum exit sites. The Plant Cell, 17, 1513–1531. 10.1105/tpc.104.026757 15805489 PMC1091771

