# Supplementary material for: Immunolocalization of hordein synthesis and transport in developing barley endosperm
Source: Plant Direct. 2024 Sep 5;8(9):e591. doi: 10.1002/pld3.591 (PMC11377179; doi:10.1002/pld3.591)
Supplement: Supplementary file 1 — Figure S1. Pileup analysis of hordeins to identify 8 family specific peptides (P1‐P8, coloured above the sequences). Repetitive sequences in the D‐hordein are underlined. Peptides 1 (B‐hor), 3 (B‐hor), 4 (B‐hor), 5 (C‐hor) did not produce MAbs which reacted to western blots. P2 gave rise to the general anti‐hordein antibody B4, used here. The ~20 amino‐acid N‐terminus indicated thus, is removed during post translational transit into the ER lumen. Table S1. Peptides used to raise 1st and 2nd round anti‐hordein monoclonals. Figure S2. Reproduced with permission from Figure 2 (Tanner et al., 2019). Hordein Accumulation in Developing Barley Grains. Frontiers in Plant Science, DOI: 10.3389/fpls.2019.00649. A. Fresh weight of developing barley grains and B. Hordein content by ELISA, calibrated against total Sloop hordein (mg/gFWt●, left y‐axis), or g/100 g protein (■, right y‐axis). Means (n = 4) ± SE are shown; no error bars are shown when SE < symbol size. Figure S3. Reproduced with permission from Figure 3, Tanner et al “Hordein Accumulation in Developing Barley Grains”. Frontiers in Plant Science, DOI: 10.3389/fpls.2019.00649. A, mid‐stage accumulation of Serpin Z4: (a), serpin Z7 (47.3 kDA), (b), serpin Z4 (44.3 kDa), (c) serpin Z4 minus 4 kDa active loop (41.6 kDa). 1Ab rabbit anti‐Z4 V6175; 2Ab Amersham (GE Heath Aust) antirabbit‐HRP. B, late‐stage accumulation mature LTP (9.0 kDa) (f). 1Ab rabbit anti‐LTP V6177, 2Ab Amersham (GE Health Australia) antirabbit‐HRP. C, early‐stage accumulation of D‐hordein (93.9 kDa) (g), C‐hordeins (70.5, 63.7 and 55.6 kDa) (h, i, j), B‐hor (47.8 kDa) (k), partly obscuring γ‐1‐hordein (45.0 kDA) (l), γ‐2‐hor (40.0 kDa) (m), γ‐3‐hordein (38.0 kDa) (n). 1Ab Sigma anti‐gluten HRP (the same pattern was observed for mouse anti‐hordeins B4, and 23‐3 with 2 Ab Amersham (GE Health Australia) anti‐mouse‐HRP). Figure S4. The original unannotated image that is shown in Figure 10. For clarity all annotations have been removed. Figure S5. Another [file PLD3-8-e591-s002.docx]

**Supplementary Material**

**Supplementary Fig. S1**: Pileup analysis of hordeins to identify 8 family specific peptides (P1-P8, coloured above the sequences). Repetitive sequences in the D-hordein are underlined. Peptides 1 (B-hor), 3 (B-hor), 4 (B-hor), 5 (C-hor) did not produce MAbs which reacted to western blots. P2 gave rise to the general anti-hordein antibody B4, used here. The ~20 amino-acid N-terminus indicated thus, is removed during co-translational transit into the ER lumen.

**| | | | | | | |80**

**C HORDEIN -MKTFLTFVLLAMAMSIVTTA----------------------------------------------------------R**

**B-HORDEIN -MKTFLIFALLVIAATSTIAQ----------------------------------------------------------Q**

**B1-HORDEIN -MKTFLIFALLAIAATSTIAQ----------------------------------------------------------Q**

**B3-HORDEIN -MKTFLIFALLAIAATNTIAQ----------------------------------------------------------Q**

**GAMMA-HORDEI -MKILIILTILAMATTFATSE----------------------------------------------------------M**

**GAMMA-HORDEI ITTTTMQFNPSGLELER---P----------------------------------------------------------Q**

**GAMMA-HORDEI -------------------------------------------------------------------------------M**

**D-HORDEIN**  **MAKRLVLFVAVIVALVALTTAEREINGNNIFLDSRSRQLQCERELQESSLEACRRVVDQQLVGQLPWSTGLQMQCCQQLR**

**| | | | | | | |160**

**SHQELQSP------------------------------------------------------QQPF (P6)**

**C HORDEIN QLNPSHQELQSP------------------------------------------------------QQPFLKQQSYLQQP**

**B-HORDEIN QPFPQ-QPF--P------------------------------------------------------QQP---------QP**

**B1-HORDEIN QPFPQ-QPI--P------------------------------------------------------QQP---------QP**

**B3-HORDEIN QPF--------P------------------------------------------------------QQP---------QP**

**GAMMA-HORDEI QVNPSVQVQ--P------------------------------------------------------TQQ---------QP**

**GAMMA-HORDEI QLFPQWQPL--P------------------------------------------------------QQP---------PF**

**GAMMA-HORDEI QVNPSVQVQ--P------------------------------------------------------TQQ---------QP**

**D-HORDEIN**  **DVSPECRPVALSQVVRQYEQQTEVPSKGGSFYPGGTAPPLQQGGWWGTSVKWYYPDQTSSQQSWQGQQGYHQSVTSSQQP**

**| | | | | | | |240**

**T---------------------------IPQQPQPYPQQ(P1)**

**C HORDEIN ------------YPQ---QPY--LPQQPFPTPQQFFPYLPQQT--------------FPPSQQPN--PLQPQQPFPLQPQ**

**B-HORDEIN ------------YPQQP-QPY--P-QQPFQ-PQQPFPQQT---------------------------IPQQPQPYPQQPF**

**B1-HORDEIN ------------YPQQP-QPY--P-QQPFP-PQQPFPQQP---------------------------VPQQPQPYPQQPF**

**B3-HORDEIN ------------YPQQP-QPY--P-QQPFP-PQQP---------------------------------------------**

**GAMMA-HORDEI ------------YPESQ-QPFISQSQQQFPQPQQPFPQQPQQP--------------FPQSQQQCLQQPQHQFPQPTQQF**

**GAMMA-HORDEI ------------LQQEPEQPY--PQQQPLP-QQQPFPQQP---------------------------QLPHQHQFPQQ--**

**GAMMA-HORDEI ------------YPESQ-QPFISQSQQQFPQPQQPF--------------------------------------------**

**D-HORDEIN**  **GQGQQGSYPGSTFPQQPGQGQQPGQRQPWSYPSATFPQQPGQGQGQQGYYPGATSLLQPGQGQQGPYQSATSPQQPGQGQ**

**| | | | | | | |320**

**TP-------------QQTPLPQGQL (P3)**

**PSTTSPQQSGQG (P9)**

**PPFGLQ-QPILS------Q (P4)**

**PLQAQQP**

**C HORDEIN PPQQPFPQPQQPNPQQPQQPFPRQPQQIVPQQPQQPFPQQPQQP------FPQPQQPFSWQPQQPFLQPLQLGPLQAQQP**

**B-HORDEIN PPQQEFPQ---------QPPF--WPQQPFPQQPPFGLQ-QPILS------QQQPCTP-------------QQTPLPQGQL**

**B1-HORDEIN PPQQPFPQ---------QPPF--WQQKPFPQQPPFGLQ-QPILS------QQQPCTP-------------QQTPLPQGQL**

**B3-HORDEIN -----FPQ---------QPPF--W------------WQ-QPVQS------QQQPCQQ-------------QQTPLPQGQQ**

**GAMMA-HORDEI PQRPLLPF---------THPFLTFPDQLLPQPPHQSFP-QPPQS------YPQPPL--------------QPFPQPPQQK**

**GAMMA-HORDEI LPQQQFPQ---------QMPL--QPQQQFPQ------Q-MPLQP------QQQPQFP-------------QQKPF--GQY**

**GAMMA-HORDEI PQRPLLPF---------THPFLTFPDQLLPQPPHQSFP-QPPQS------YPQPPL--------------QPFPQPPQQK**

**D-HORDEIN**  **GQQEPYPIATSPH----QPGQ--WQQPGQGQQGYYPSVTSPQQSGQGQQGYPSTTSPQQSGQGQQLGQGQQPGQGQQGYP**

**| | | | | | | |400**

**FPLQP (P5) PSTTSPQQSGQG (P9)**

**C HORDEIN FPLQPQLPFPQPQ----QPIGQQPKQPLLQQ--PQQTIPQQPQQ------------PFPLQPQQPFP-------------**

**B-HORDEIN YQTLLQLQIPYVH----PSILQ--------QLNPCKVFLQQQCS------------PVRM-PQLIA--------------**

**B1-HORDEIN YQTLLQLQIQYVH----PSILQ--------QLNPCKVFLQQQCS------------PVPV-PQRIA--------------**

**B3-HORDEIN YQPLLQQQIPFVH----PSVLQ--------QLNPCKVFLQQQCS------------PVPM-PQRIA--------------**

**GAMMA-HORDEI YPEQPQQPFPWQQ----PTIQL----YLQQQLNPCKEFLLQQCR------------PVSL-LSYIW--------------**

**GAMMA-HORDEI QQPLTQQPYPQQQ----PLAQQQPSIEEQHQLNLCKEFLLQQCTLDE---------KVPL-LQSVIS-------------**

**GAMMA-HORDEI YPEQPQQPFPWQQ----PTIQL----YLQQQLNPYKEFLLQQCR------------PVSL-LSYLW--------------**

**D-HORDEIN**  **SATFPQQPGQWQQGSYPSTTSPQQSGQGQQGYNPSGTSTQQPGQVQQLGQGQQGYYPIATSPQQPGQGQQLGQGQQPGHG**

**| | | | | | | |480**

**C HORDEIN --------------------------------QQPQQPLPQQPQQIIS-------------QQPQQPFPLQPQ-------**

**B-HORDEIN ----------------------------------RLQMLQQSSCHVLQ-------------QQCCQQLPQISE-------**

**B1-HORDEIN ----------------------------------RSQMLQQSSCHVLQ-------------QQCCQQLPQIPE-------**

**B3-HORDEIN ----------------------------------RSQMLQQSSCHVLQ-------------QQCCKQLPQIPE-------**

**GAMMA-HORDEI -----------------------------------SKIVQQSSCRVMQ-------------QQCCLQLAQIPE-------**

**GAMMA-HORDEI --------------------------------FLRPHISQQNSCQLKR-------------QQCCQQLANINE-------**

**GAMMA-HORDEI -----------------------------------SKIVQQSSCRVML-------------QQCCLQLAQIPE-------**

**D-HORDEIN**  **QQLVQGQQQGQGQQGHYPSMTSPHQTGQGQKGYYPSAISPQQSGQGQQGYQPSGASSQGSVQGACQHSTSSPQQQAQGCQ**

**| | | | | | | |560**

**C HORDEIN -------------------------------------------------------QPFPQP-------Q-----------**

**B-HORDEIN -------------------------------------------------------Q-FRHEAIRAIVYS-----------**

**B1-HORDEIN -------------------------------------------------------Q-FRHEAIRAIVYS-----------**

**B3-HORDEIN -------------------------------------------------------Q-FRHEAIRAIIYS-----------**

**GAMMA-HORDEI -------------------------------------------------------Q-YKCTAIDSIVHA-----------**

**GAMMA-HORDEI -------------------------------------------------------Q-SRCPAIQTIVHA-----------**

**GAMMA-HORDEI -------------------------------------------------------Q-YKCTAIDSIVHA-----------**

**D-HORDEIN**  **ASSPKQGLGSLYYPSGAYTQQKPGQGYNPGGTSPLHQQGGGFGGGLTTEQPQGGKQPFHCQQTTVSPHQGQQTTVSPHQG**

**| | | | | | | |640**

**TQQQLQQEQVGQ (P2)**

**ESEQIIT---QQPFP (P8)**

**C HORDEIN ----PFPQE-------------QPQQAFPLQPQQPFPEESEQIIT---QQPFPLQPQQ----LFPQQP------QQPLPQ**

**B-HORDEIN ----IFLQE-------------QPQQSVQGVSQTQQQLQQEQVGQCSFQQPQPQQLG-----QAQQVP------QSVFLQ**

**B1-HORDEIN ----IFLQE-------------QPQQLVEGVSQPQQQLWPQQVGQCSFQQPQPQQVG-----QQQQVP------QSAFLQ**

**B3-HORDEIN ----IILQEQQQVQDFVQPQQQQPQQSVQGVSQSQQQSQQPQLGQCSFQQPQLQQLGQ--QPQQQQVP------LWAFLQ**

**GAMMA-HORDEI ----IFMQQ-------------GQRQGV-------------QIVQ---QQPQPQQVG-----QCVLVQ------GQGVVQ**

**GAMMA-HORDEI ----IVMQQ-------------QVQQ---------------QVGH-GFVQSQLQQLGQGMPIQLQQQP------GQAFVL**

**GAMMA-HORDEI ----IFMQQ-------------GQRQGV-------------QIVQ---QQPQPQQVG-----QCVLVQ------GQGVVQ**

**D-HORDEIN**  **QQTTVSPHQGQQTTVSPH----QGQQTTVSPHQGQQTTVSPHQGQQTTVSPHPGQQTTVSPHQGQQTTVSPHPGQQTTVS**

**| | | | | | | |720**

**KYIIPQQPQQPF (P7)**

**C HORDEIN PQQ-------PFRQLPKYIIPQQPQQPFLL---------QPHQPQQ----PYAQQDIWS---------------------**

**B-HORDEIN PHQ-----IAQLEATTSIALRTLPRMCNVN---------VPLYDIM----P---PDFWH---------------------**

**B1-HORDEIN PHQ-----IAQLEATTSIALRTLPMMCSVN---------VPLYRIL----RGVGPSVGV---------------------**

**B3-HORDEIN PQQ-----MAQLEVMTSVALRTLPTMCNVN---------VPLYGIT----TSVPLSVGT---------------------**

**GAMMA-HORDEI PQQ-----LAQMEAIRTLVLQSVPSMCNFN---------VPPN-CSTIKAPFVGVVTGV---------------------**

**GAMMA-HORDEI PQQ-----QAQFKVVGSLVIQTLPMLCNVH---------VPPY-CS----PFGSMATGS---------------------**

**GAMMA-HORDEI PQQ-----LAQMEAIRTLVLQSVPSMCNFN---------VPPN-CSTIKAPFVGVVTGV---------------------**

**D-HORDEIN**  **PHQGQQTTVSPHQGQQTTVSPHQGQQTTVSPHQGQQTTVSPHQGQQTTVSPHQGQQPGEQPCGFPGQQTTVSLHHGQQSN**

**| | | | | | | |800**

**C HORDEIN ---------------------------------------DIALLG--**

**B-HORDEIN -----------------------------------------------**

**B1-HORDEIN -----------------------------------------------**

**B3-HORDEIN ---------------------------------------GVGPY---**

**GAMMA-HORDEI ---------------------------------------GGQ-----**

**GAMMA-HORDEI ---------------------------------------GGQ-----**

**GAMMA-HORDEI ---------------------------------------GGQ-----**

**D-HORDEIN ELYYGSPYHVSVEQPSASLKVAKAQQLAAQLPAMCRLEGGGGLLASQ**

**Supplementary Table S1:** Peptides used to raise 1^st^ and 2^nd^ round anti-hordein monoclonals.

| Peptide Identifier (monoclonal clones) | Targeted hordein family | Initial Peptide  Sequence | Useful monoclonal clones identified in first round western screening (Hordein family identified) | Second round modified PQ poor sequence (none were useful) |
| --- | --- | --- | --- | --- |
| P1 (B1, B7) | B | TIPQQPQPYPQQ | no |  |
| P2 (B4) | B | TQQQLQQEQVGQ | B4 (C, B, γ_3_,) | QEQVGQCSF |
| P3 (B2) | B | TPQQTPLPQGQL | no |  |
| P4 (B3, B5, B6) | B | PPFGLQQPILSQ | no |  |
| P5 (C1) | C | PLQAQQPFPLQP | no |  |
| P6 (C3, C5) | C | SHQELQSPQQPF | C3 (B, γ_1,3)_ | SHQELQS |
| P7 (C4, C6, C7, C8) | C | KYIIPQQPQQPF | C8 (C, B, γ_1,2,3_) | FRQLPKYIIP |
| P8 (C2) | C | ESEQIITQQPFP | C2 (γ_3)_ | PEESEQIIT |
| P9 (D1, D2) | D | PSTTSPQQSGQG | D1 and D2  (B, γ_1,2,3)_ | GSYPSTTSP |


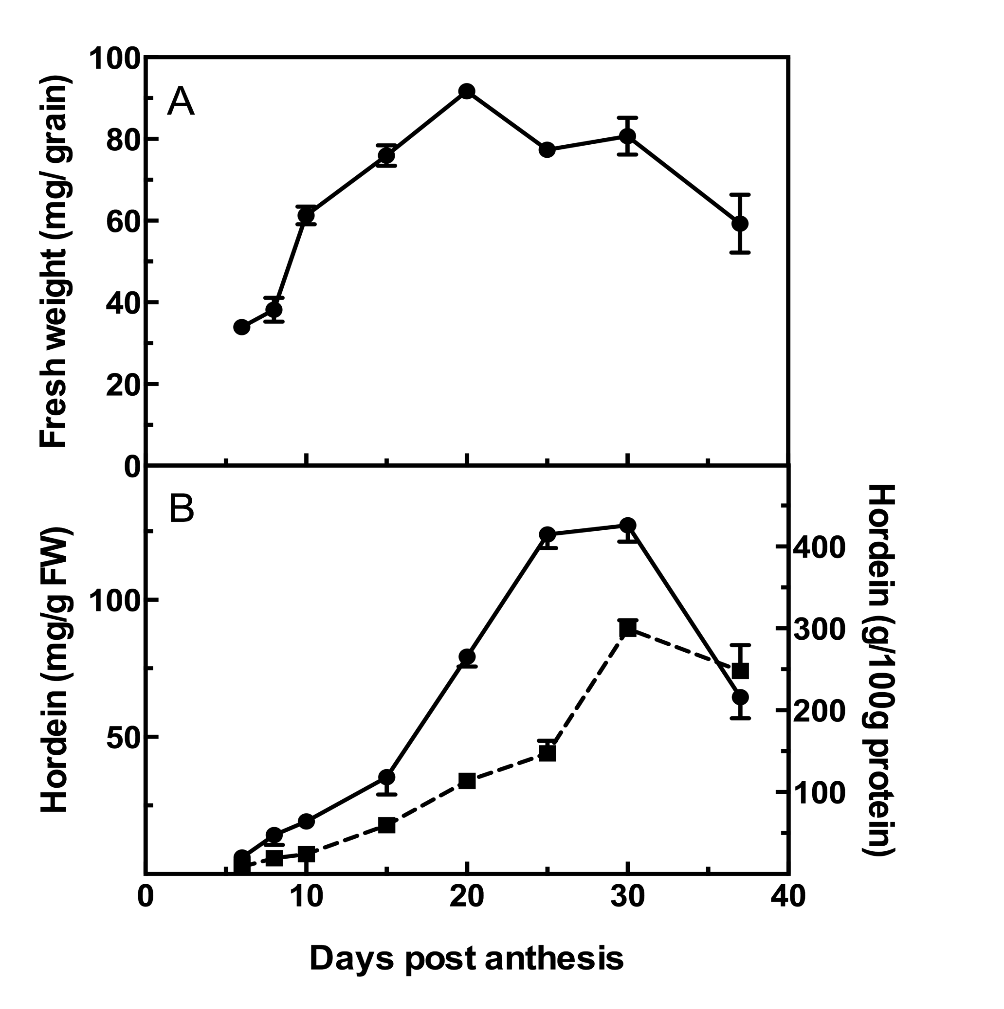


**Supplementary Fig. S2.** Reproduced with permission from Fig 2, (Tanner *et al.*, 2019). Hordein Accumulation in Developing Barley Grains. Frontiers in Plant Science, DOI: 10.3389/fpls.2019.00649. A. Fresh weight of developing barley grains and B. Hordein content by ELISA, calibrated against total Sloop hordein (mg/gFWt⏺, left y-axis), or g/100g protein (⏹, right y-axis). Means (n=4) ± SE are shown; no error bars are shown when SE< symbol size.

**Supplementary Fig. S3**. Reproduced with permission from Fig 3, Tanner et al “Hordein Accumulation in Developing Barley Grains”. Frontiers in Plant Science, DOI:10.3389/fpls.2019.00649.

A, mid-stage accumulation of Serpin Z4: (*a*), serpin Z7 (47.3 kDA), (*b*), serpin Z4 (44.3 kDa), (*c*) serpin Z4 minus 4 kDa active loop (41.6 kDa). 1Ab rabbit anti-Z4 V6175; 2Ab Amersham (GE Heath Aust) antirabbit-HRP.

B, late-stage accumulation mature LTP (9.0 kDa) (*f*). 1Ab rabbit anti-LTP V6177, 2Ab Amersham (GE Health Australia) antirabbit-HRP.

C, early-stage accumulation of D-hordein (93.9 kDa) (*g*), C-hordeins (70.5, 63.7 and 55.6 kDa) (*h, i, j*), B-hor (47.8 kDa) (*k*), partly obscuring $\gamma$-1-hordein (45.0 kDA) (*l*),

$\gamma$-2-hor (40.0 kDa) (*m*), $\gamma$-3-hordein (38.0 kDa) (*n*). 1Ab Sigma anti-gluten HRP (the same pattern was observed for mouse anti-hordeins B4, and 23-3 with 2 Ab Amersham (GE Health Australia) anti-mouse-HRP).

The relative molecular weights (given in parentheses, in kDa) were determined by calibration against Invitrogen pre-stained standards, which were in turn calibrated against Invitrogen unstained standards.


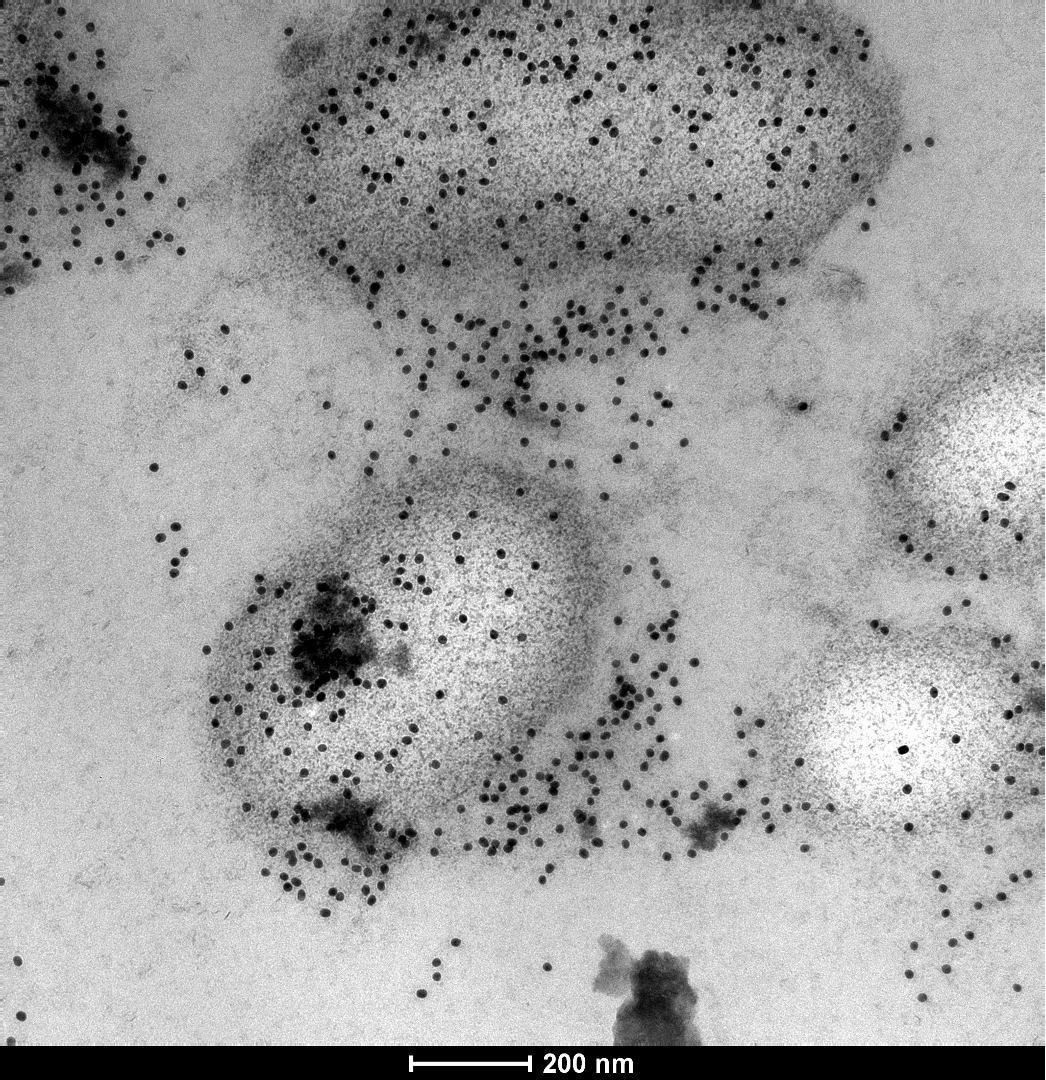


**Supplementary Fig. S4.** The original unannotated image that is shown in Fig. 10. For clarity all annotations have been removed.


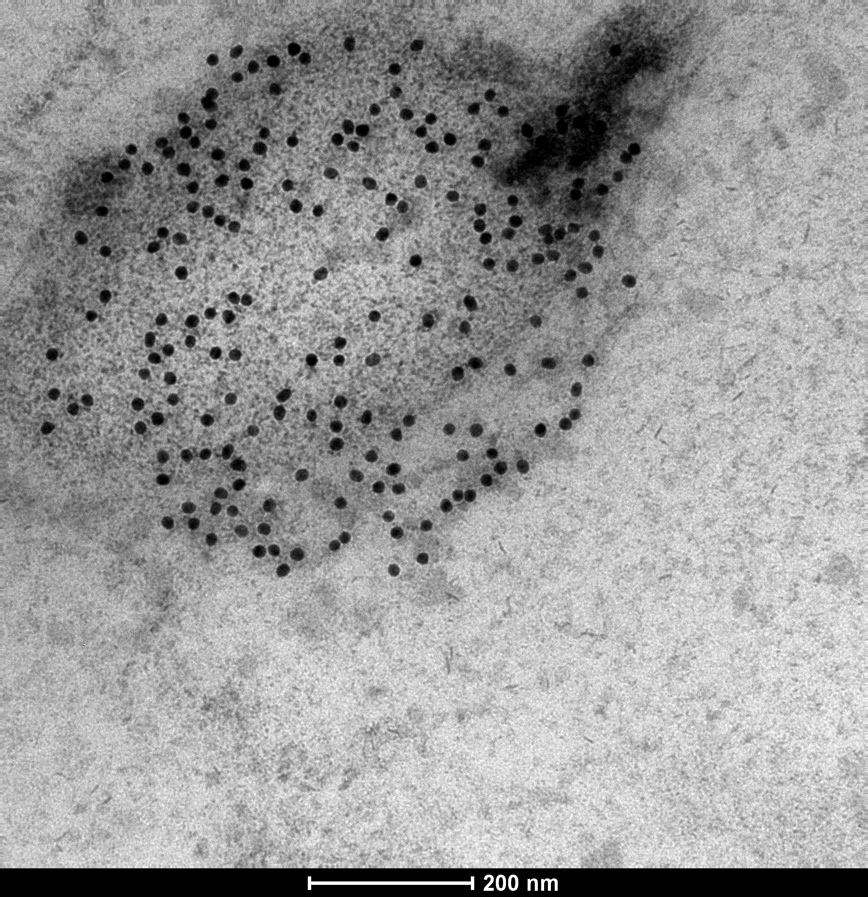


**Supplementary Fig. S5**. Another example of hordeins outside a protein body, same section as Fig 10.


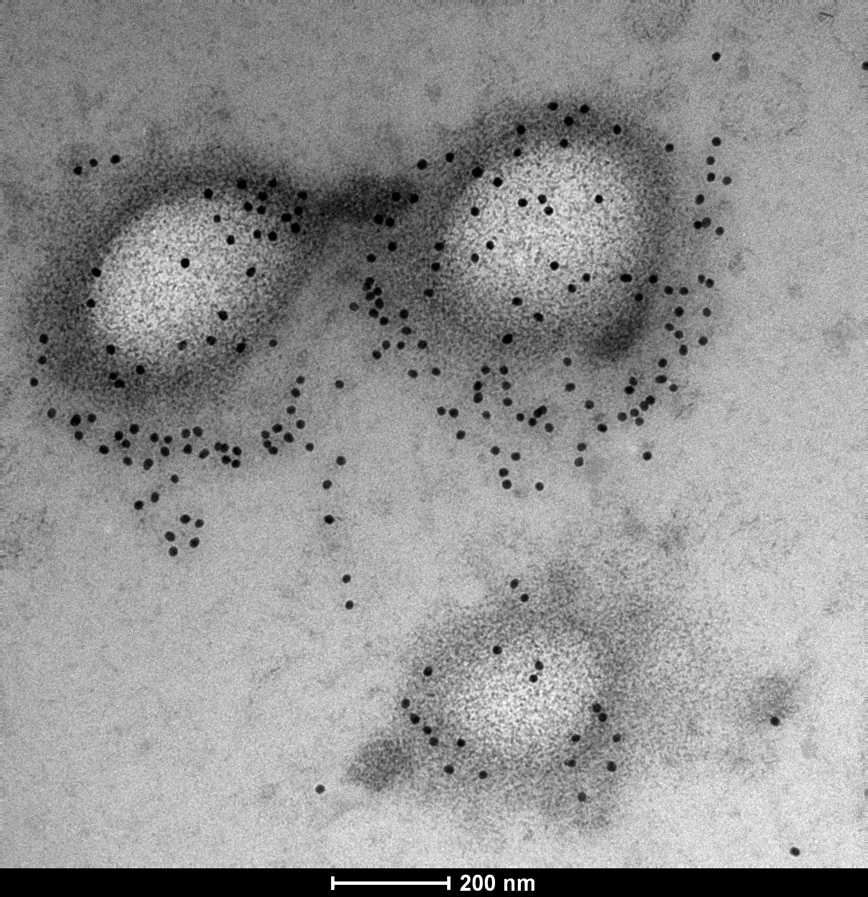


**Supplementary Fig. S6**. Another example from the same section as Fig 10.


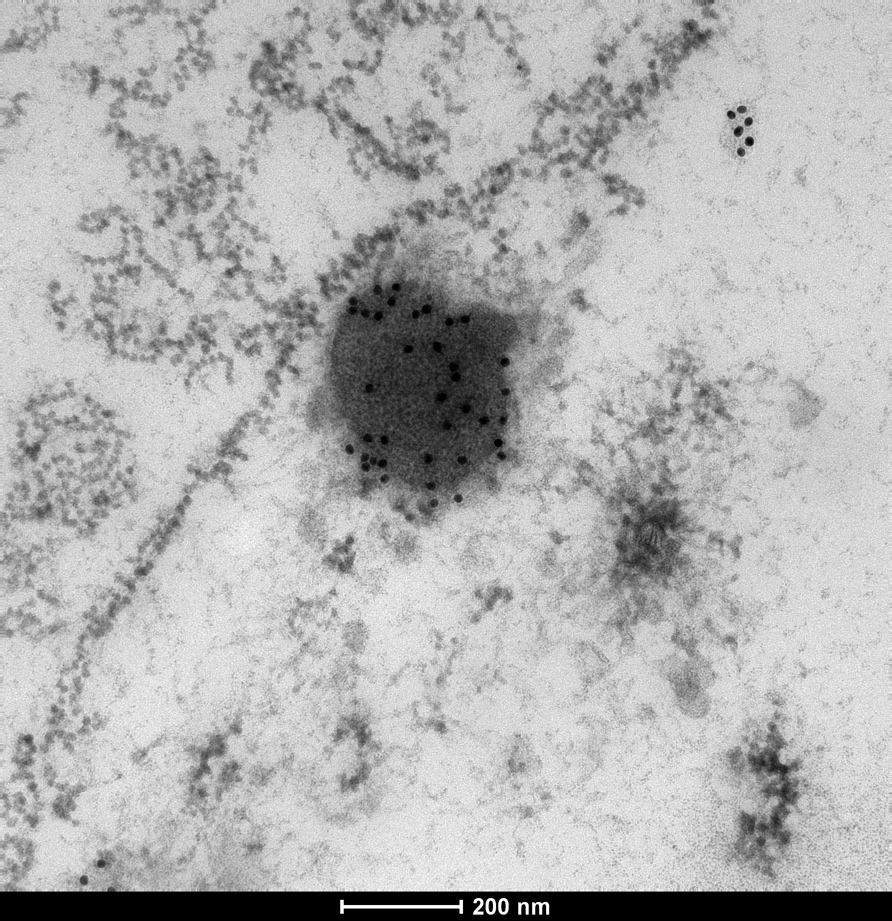


**Supplementary Fig. S7.** Another example of hordeins outside PB from a different section , same block to Fig 10.


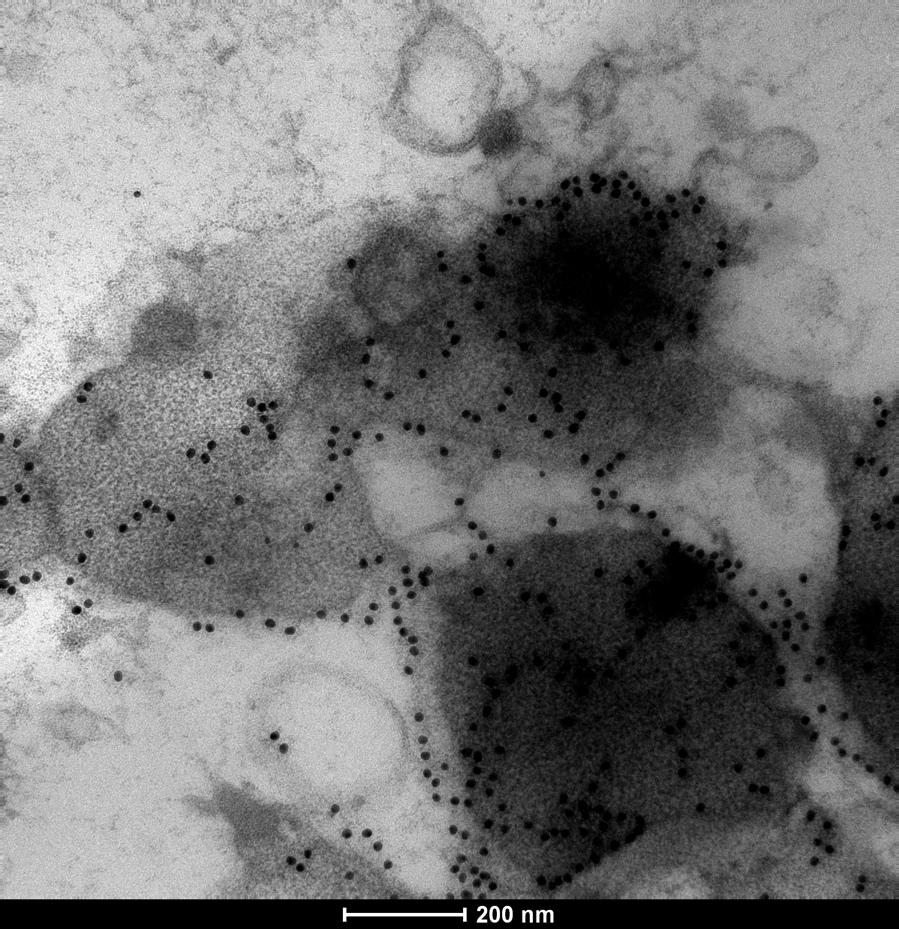


**Supplementary Fig. S8.** Another example of hordeins outside PB from a different section, same block to Fig 10.


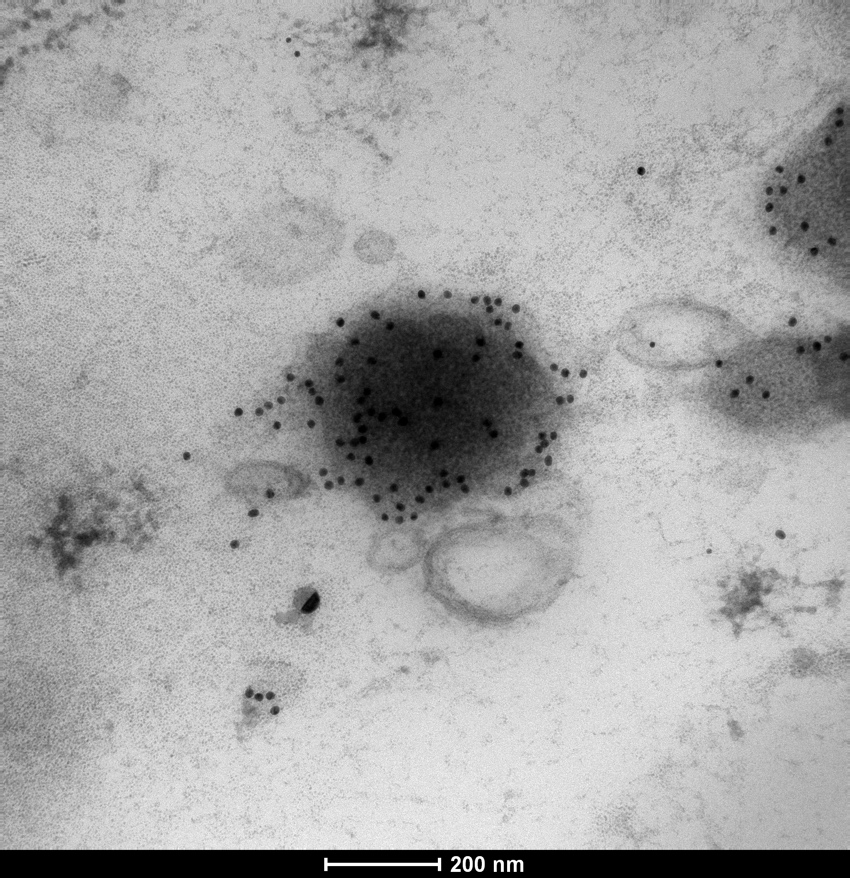


**Supplementary Fig. S9.** Another example of hordeins outside PB, from a different section, same block to Fig 10.


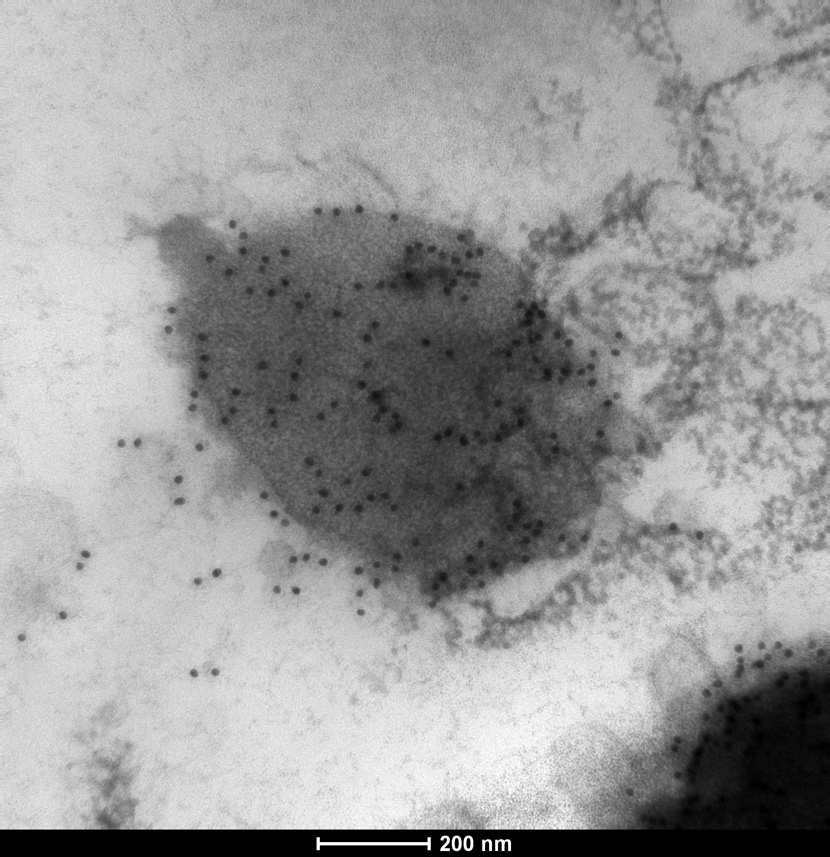


**Supplementary Fig. S10.** Another example of hordeins outside PB, from a different section, same block as Fig 10.

**Supplementary Table S2. Proportion of labelled hordeins outside the limits of the protein body.**

| Figure No. | Number of hordeins inside the PB | Number of hordeins in second area but outside the PB | Isolated in Cytsol | % of total hordein outside protein body |
| --- | --- | --- | --- | --- |
| 9 | 285 | 161 | 10 | 35.3 |
| 11B | 148 | 70 | 4 | 31.5 |
| 11D | 176 | 119 | 5 | 39.5 |
| S5 | 117 | 53 | 0 | 31.2 |
| S6 | 50 | 153 | 22 | 68 |
| S7 | 26 | 13 | 6 | 28.9 |
| S8 | 255 | 147 | 6 | 57.7 |
| S9 | 80 | 50 | 12 | 35.2 |
| S10 | 78 | 49 | 14 | 62.8 |
| Mean | 135 | 90.6 | 8.8 | 43.3 |
|  |  |  | STDEV | 14.3 |
|  |  |  | SE | 4.5 |

The percentage of hordeins that lay outside the limits of the protein body, but within the second area of increased granularity relative to the cytoplasm which was closely associated with the protein body varied from 31.2-68.0 % (Mean + SE 43.3 + 4.5) **Fluorescence Controls:**


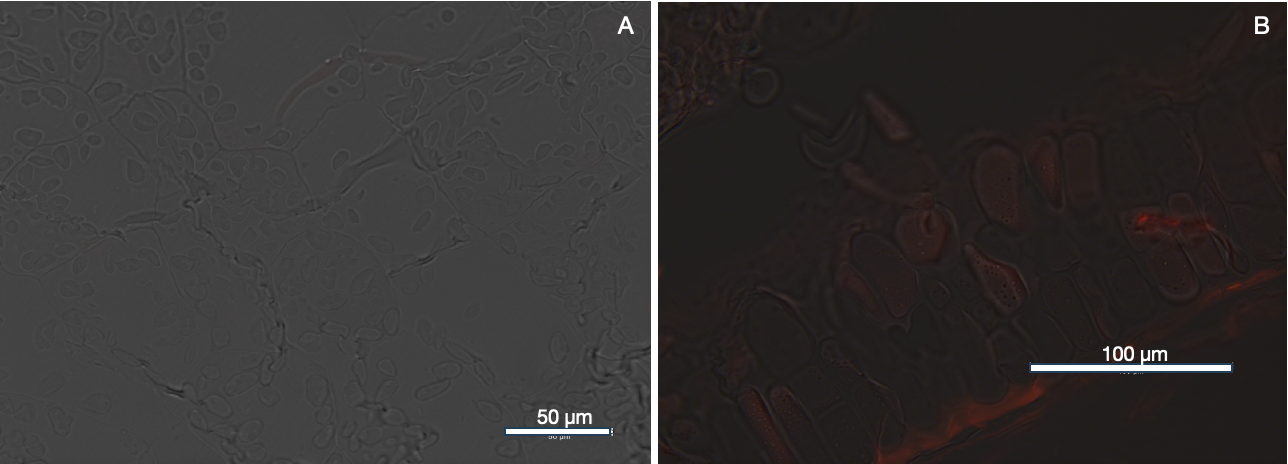


**Supplementary Fig. S11.** Second Ab alone control of barley cv Sloop, with Invitrogen donkey anti-mouse-IgG-Alexa Flour 568 at (A) 8 DPA, scale bar 50 µm; (B) 30 DPA, scale bar 100 µm. Some slight autofluorescence occurs at the testa/pericarp, at 5 oclock. Goat anti-rabbit IgG-Alexa Fluor 568 (Abcam) alone, also did not generate a signal but the original image was lost.

**iEM controls**


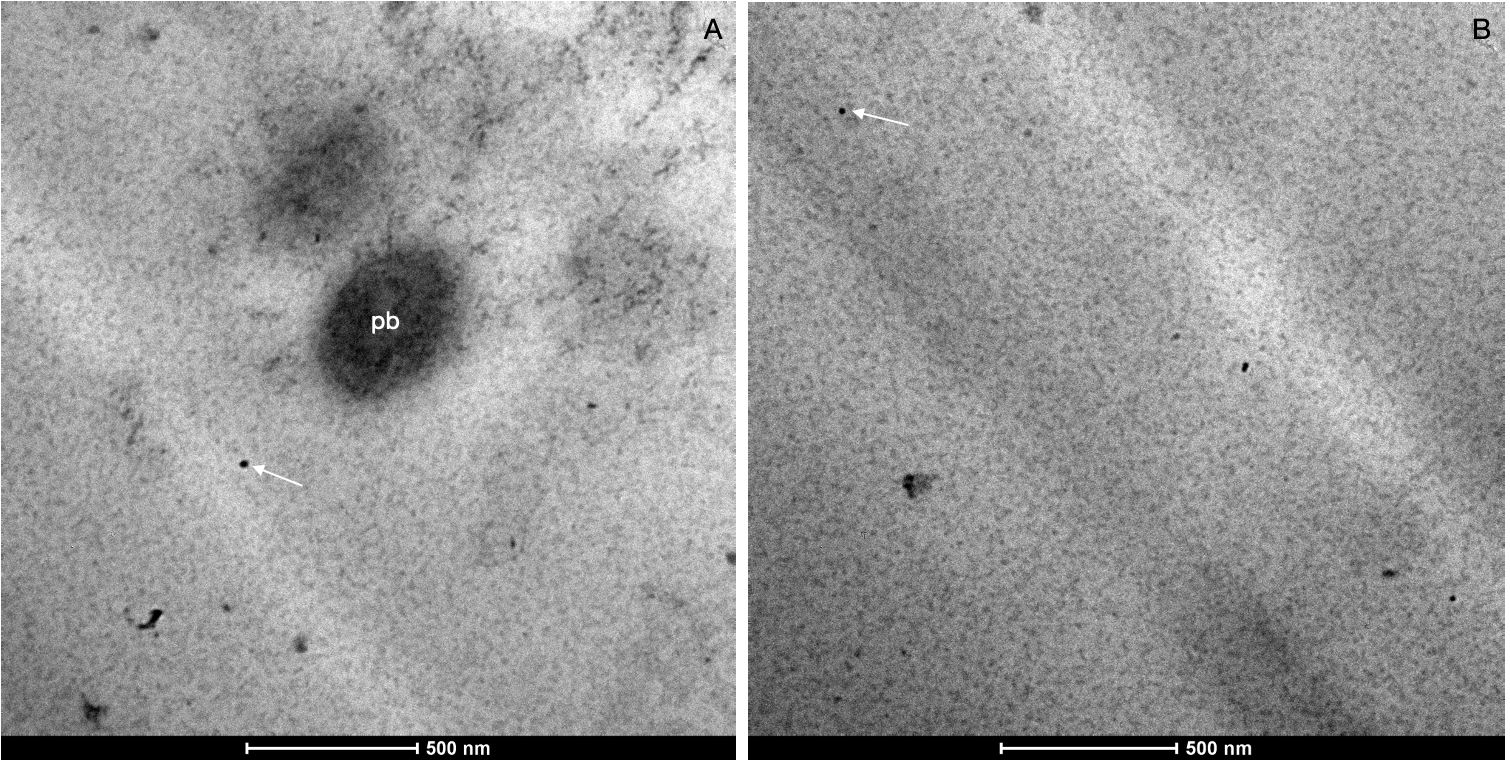


**Supplementary Fig. S12.** Second Ab control of barley cv Sloop, at 15 DAP with AR-10 nm gold (A) showing protein body; (B) showing cytoplasm, scale bars 500 nm. AR-10 nm gold shows one particle per field at this magnification (*white arrow*).


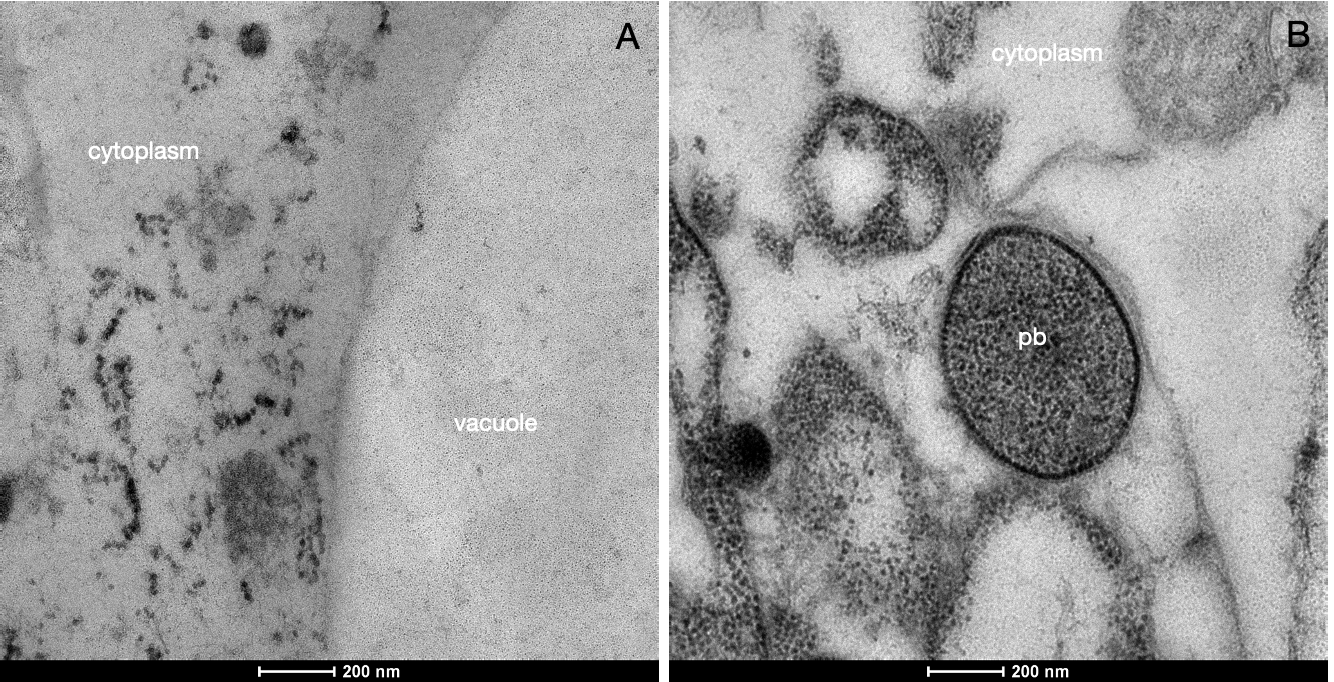


**Supplementary Fig. S13.** Second Ab control of barley cv Sloop with AR-18 nm gold at 8 DPA (A) showing unlabeled areas of cytoplasm (*cytoplasm*) and vacuole (*vacuole*); (B) unlabeled protein body (*pb*) and cytoplasm (*cytoplasm*) . Scale bars are 200 nm.


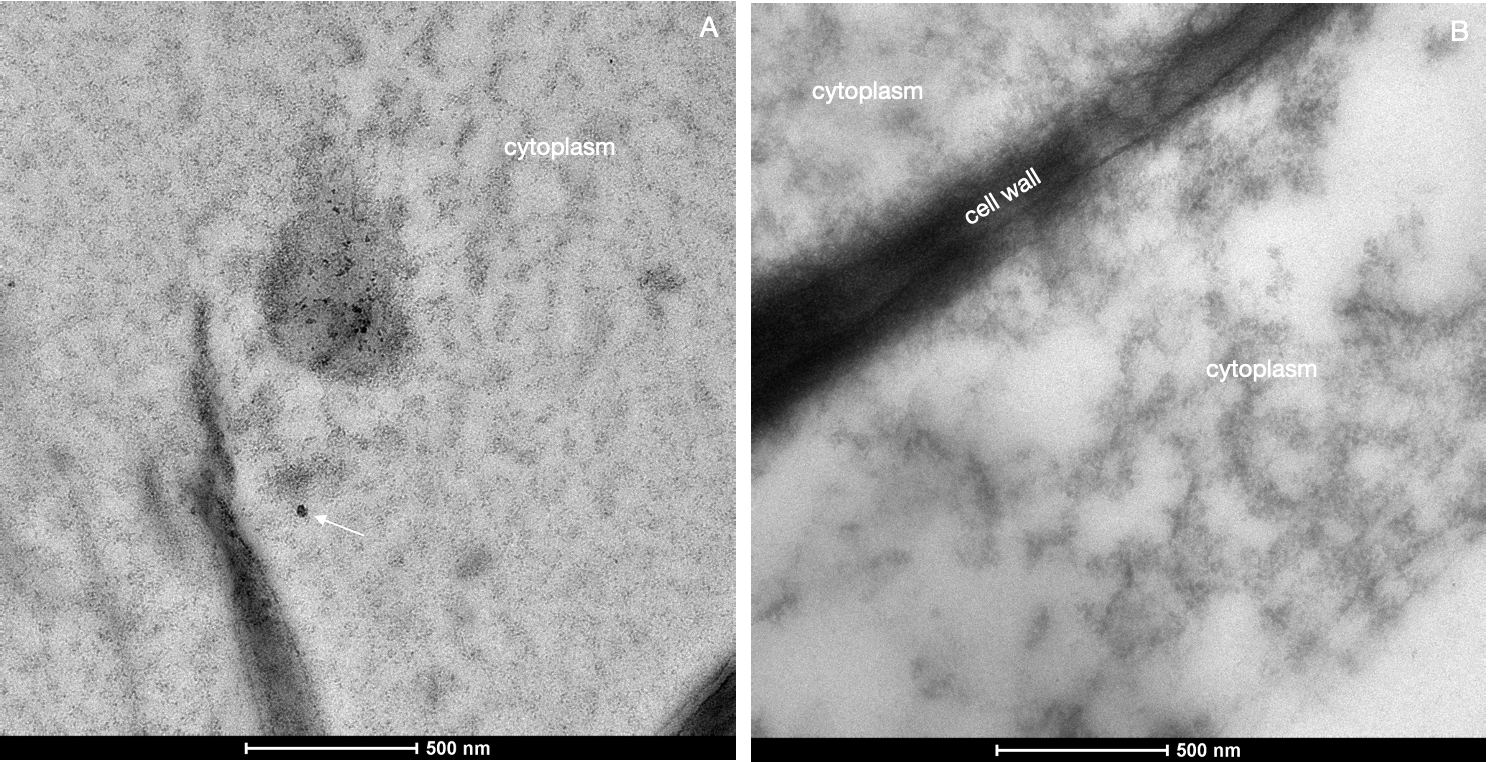


**Supplementary Fig. S14.** Second Ab control of barley cv Sloop at 10 DPA, with AM-10 nm gold (A) showing 1 particle (*white arrow*) in an area of cytoplasm (*cytoplasm*). The dark area at six o’clock is a fold in the section; (B) showing unlabeled cell wall (*cell wall*) and areas of cytoplasm (*cytoplasm*). Scale bars 500 nm.


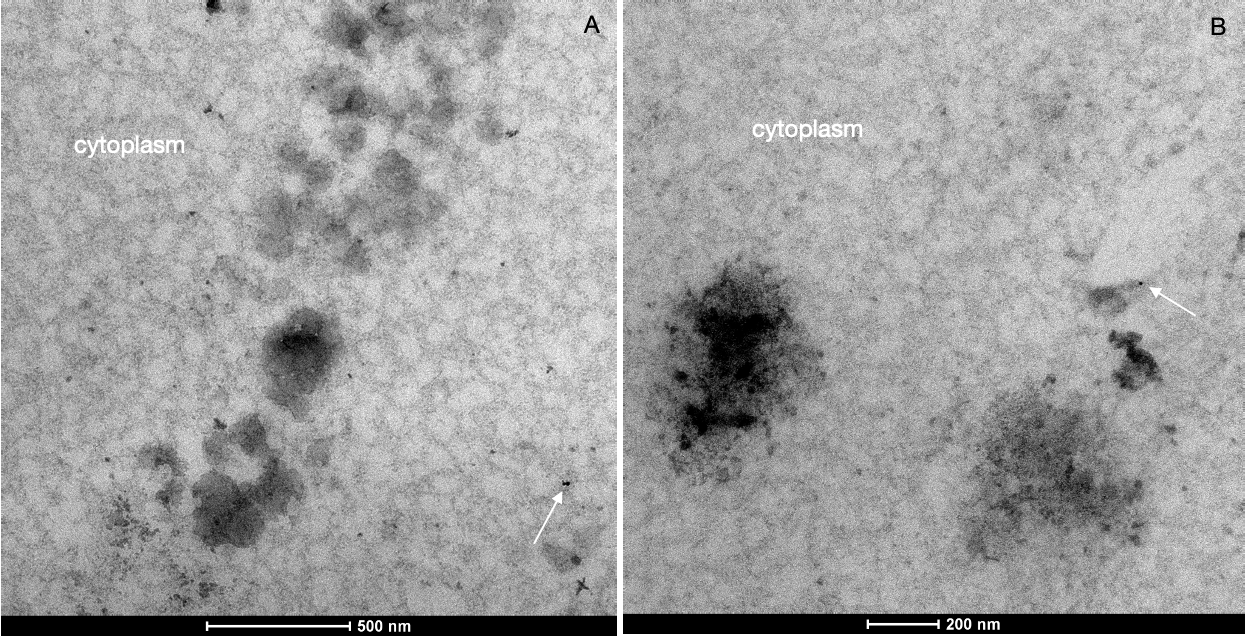


**Supplementary Fig. S15.** Second Ab control of barley cv Sloop at 15 DPA, with AM-18 nm gold (A, B) each showing one particle per field (white arrows) in an area of cytoplasm (*cytoplasm*). Scale bars are 500 nm (A) and 200 nm (B).
