# Supplementary material for: Immunolocalization of hordein synthesis and transport in developing barley endosperm
Source: Plant Direct. 2024 Sep 5;8(9):e591. doi: 10.1002/pld3.591 (PMC11377179; doi:10.1002/pld3.591)
Supplement: Supplementary file 2 — Data S1. Peer Review. [file PLD3-8-e591-s001.pdf]

Plant Direct Open Access

Immunolocalization of hordein synthesis and transport in developing barley endosperm.

Running title: Hordein intracellular transport

Gregory Tanner[1], Allison van de Meene[1,2], Anthony Bacic[1,3]

Decision Letter Round 1:

November 29, 2023

Dr. Gregory J Tanner
University of Melbourne, Melbourne, Australia
Melbourne, N/A
Australia

RE: Immunolocalization of hordein synthesis and transport in developing barley endosperm.

Dear Dr. Tanner:

Thank you for submitting to Plant Direct. All required reviews have been returned and we have now finished our evaluation of your manuscript. In light of the reviewers' and editor's comments, further revisions are needed before the paper can be accepted for publication in Plant Direct.

Please view the editors' and reviewers' comments below and use their suggestions as a guide while you work on your revision.

When uploading the revised version of this article, please be sure to include the following:

-A word document that contains your response to the reviewers. You should respond to each reviewer comment and note the changes made to the manuscript. If you do not agree with a reviewer's comment and choose not to make a suggested revision, please explain why. Please try to provide as complete an answer as possible to each reviewer's criticisms.
-A tracked changes document with each change highlighted
- A clean version of the latest version of the manuscript

To upload your revision, please click the link below.
https://plantdirect.msubmit.net/cgi-bin/main.plex?el=A1Lr2dv3A4oUg5I4A9ftdQk91RsV283b15KtQIWfGQwZ

In order to provide as timely a service as possible, we ask that your revision is resubmitted within three months after receipt of this request. If an extension is needed, please send a request, along with a brief explanation, to the editorial office at plantdirect@wiley.com .

Please note that, in addition to publishing reviewer comments, the author's responses to review comments will also be published alongside the final version of the paper. If you would not like the author's responses to be published, please contact the editorial office at plantdirect@wiley.com .

Thank you very much for giving us an opportunity to review your work. I look forward to receiving the next version.

Sincerely,

Hsou-min Li

Editor, Plant Direct

-- Reviewer comments:
Reviewer #1:

This is an interesting study on the subcellular localization of hordein in developing barley endosperm via both immubofluorescent and immunogold-TEM approaches, in which HPF was used to fix the samples for TEM analysis. The quality of most TEM images is acceptable. Overall, the TEM data may provide some useful information for future research in the field.

The speculation "one pathway directly via exocytosis from ER lumen" may not be correct, as "exocytosis" refers to "reaching outside of the cell", rather, the authors may refer "ER protein export" either via COPII vesicles or direct ER to PSV pathway.

Other suggestions for improvement:

1. The term "exocytosis or endocytosed" used in the MS needs to be revised according to the current knowledge in the field (e.g. endocytosis, exocytosis, invagination, engulfment, transport vesicles, membrane fusion).

2. Most of the text labelings in the TEM images are too large or not position properly (that mess the structures): better format of arrows should be used.
3. Controls for immunogold-TEM should be provided to demonstrate specific labelling in this study.
4. A discussion on the limitation of fixed samples for iEM should be included vs. live-cell imaging for making conclusions of transport pathways (Figure 13 and title).
5. A summary on the major findings from this study vs. previous studies would be helpful for justifying the novelty of this study.
6. Some speculations should be backed up by suggestion on future research.
7. Most Figures (2,3,4,7,8,9,10,11) should be modified so that individual images be clearly separated (as shown in Figure 6).

Reviewer #2:

These authors carried out extensive cell biology studies, at both light and electron microscopic levels and also with immunogold labeling, to investigate the processing and packaging of barley endosperm storage proteins. This topic has been investigated frequently in the past in cereals and many papers have been published. The current state of knowledge indicates that cereal seed storage proteins are initially synthesized on ER followed by processing and transport to protein bodies for storage. The manuscript is thoroughly written in the intro and methods sections, but kind of brief in the discussion, which is unfortunate because there appear to be a lot of issues need to be discussed. The authors have presented a good number of micrographs serving as the key experimental evidence in support of the model/process shown in Fig 13. However, the quality (particularly the resolution) of some micrographs is not good enough to be convincing.

While the work does not contain breakthrough results, a couple of unique features are presented:
A. Hordeins and two other storage proteins, serpin Z4 and LTP1, are likely processed in the same route, ending up being stored in vacuoles.
B. The processing of these storage proteins does not traverse the Golgi Apparatus (GA).

While these unique features could further enhance our knowledge in seed storage protein processing and storage, the following points have to be adequately addressed before the final decision could be made about the suitability of publishing this work in Plant Direct.

1. Two obvious mistakes need to be corrected. First, "LTP" stands for lipid transfer protein, not lipid transferase protein (see Abstract). A name ending with "-ase" is reserved for an enzyme, such as amylase, nuclease, kinase, polymerase, etc,, and lipid carrier protein is not an enzyme because it's not involved in the formation and breakage of a covalent bond. Second, hordeins are glycosylated (for example, see Snegaroff et al (2013) BBA, 1834: 395-403). Thus, the last

sentence in the Abstract is erroneous at least for gamma-hordeins.
2. In Fig 3, the authors claimed that small vacuoles were observed (those circled). How could they rule out the possibility that these "small vacuoles" were actually protein bodies?
3. In Fig 4, the authors claimed that small protein bodies were arranged along ER (circled). Without observing ER in the same micrograph, how could they draw this conclusion?
4. Fig 5 contains a lot of potentially interesting information, yet the quality is not good enough to reveal the details (especially where those white arrows are). More importantly, what are those small vesicles on the right side of this micrography (just to the left of cell walls)?
5. In Fig 6, what are in the circled regions?
6. In Fig 7A, the authors claimed that blue arrows indicated that cytosolic hordeins were being taking up by vacuoles. There are only three gold particles in total in support of this claims. The authors need to provide much more convincing data to substantiate this potentially intriguing process.
7. In Fig 7B, the authors claimed that the black arrows indicated where ribosomes were. This is hardly convincing. Please provide micrography with higher magnification to clearly show that these dots are indeed ribosomes (see my evaluations of Fig 11 below).
8. Fig 8 reveals intriguing information, i.e., there were substantial gold particles outside protein bodies, presumably in cytosol. How could this happen, protein body rupturing or via some other mechanisms? It would be important that the authors can provide a semi-quantitative estimation about the percentage of total hordeins being cytosolic vs in protein bodies/vacuoles. If Fig 8 is a good representative figure, almost 50% of hordeins are actually cytosolic, which is quite significant. How could this much hordeins in protein bodies be released? Does this mean a lot of protein bodies are somehow ruptured along the way to final destination? Or, are there other mechanisms can account for the high percentage of hordeins being in the cytosol? Furthermore, if hordeins are water-insoluble (as indicated in the very first sentence in the Intro section), how could these cytosolic hordeins stay soluble without the protection of a membranous vesicle?
9. Also in Fig 8, the "membrane surrounding label" on the left half of the figure is not visible to the eyes of this reviewer, hence not convincing.
10. In Fig 10B, there were so many gold particles on the peripheral of a protein body. Are these hordeins be taken up by the protein body as suggested in Fig. 6? Frankly, this photo is more impressive than the two blue arrows in Fig. 6.
11. In Fig 11B, it is hard to recognize those structures pointed by the orange arrows as ribosomes. The authors need to provide more convincing micrographs.
12. In Fig 12, the GA indeed looks clear for hordein signals. Can the authors provide 1-2 more photos like this in the supplemental material section in support of the important notion that hordeins do not traverse GA in its processing?
13. Since serpin Z4 is synthesized without a signal peptide, how could this protein enter ER and then processed with hordeins in the same protein bodies as the authors have suggested?

Decision Letter Round 2:

Plant Direct
Open Access

April 25, 2024

Dr. Gregory J Tanner
University of Melbourne, Melbourne, Australia
Melbourne, N/A
Australia

MSID: 2023-01334-TWR1
MS TITLE: Immunolocalization of hordein synthesis and transport in developing barley endosperm.

Dear Dr. Gregory Tanner:

I am pleased to inform you that your manuscript "Immunolocalization of hordein synthesis and transport in developing barley endosperm." has been accepted for publication in Plant Direct.

Your article will appear online in the next available issue of Plant Direct. To ensure your article gets published as quickly as possible, please pay attention to the steps detailed below. We have found that most of the delays happen at this stage, especially at the payment stage, so please respond as quickly as possible when prompted.

License Agreement: Once your article has been accepted it will move to Production and undergo admin and file checking - you may receive an email with any queries we have at this stage. When all required items are received by the publisher and queries resolved, the corresponding author will receive an email from Wiley's Author Services system which will ask them to log on at https://authorservices.wiley.com/bauthor and will present them with the appropriate license for completion. Your article cannot be published until both the signed license agreement and payment of the article fee have been received.

Payment of the Open Access Article Publication Fee: All articles published in Plant Direct are fully open access: immediately and freely available to read, download and share. Plant Direct charges a publication fee to cover the publication costs. The corresponding author for this manuscript should have already received a quote from the payments team (cs-openaccess@wiley.com) with the estimated article publication fee; please email cs-openaccess@wiley.com if this has not been received. The corresponding author should log on to the Wiley Author Services site, where the publication fee can be paid by credit card or an invoice. Pro Forma can also be requested. Payment of the publication charge must be received before the article will be published online.

Proofs: You will have the opportunity to look over your paper once more when you receive the author proofs for your article. The proofs will be with you in approximately two weeks. Please note that, in addition to publishing reviewer comments, the author's responses to review comments will also be published alongside the final version of the paper. If you would not like

the author's responses to be published, please contact the editorial office at plantdirect@wiley.com .

Promotion of your article: You can help your research get the attention it deserves! Check out Wiley's free Promotion Guide for best-practice recommendations for promoting your work at www.wileyauthors.com/eeo/guide. And learn more about Wiley Editing Services which offers professional video, design, and writing services to create shareable video abstracts, infographics, conference posters, lay summaries, and research news stories for your research at www.wileyauthors.com/eeo/promotion.

Thank you again for your contribution to Plant Direct. If you have any questions, feel free to contact the editorial office at plantdirect@wiley.com .

Sincerely,

Esther van der Knaap

Esther van der Knaap

Editor, Plant Direct

--- Editor comments

--- Reviewer comments:
Reviewer #1:

I do see improvement in this revised MS.
However, 1) no responses/changes to address comments in the file; 2) the authors did not address my previous comments on most Figures (now Figures 2,3,4,7,8,9,10,11) in which individual images should be clearly separated from each other as shown in Figure 6.

Reviewer #2:

This reviewer appreciates that authors have taken all points raised in the review seriously and addressed them thoroughly with corrections and revisions. The revised ms is acceptable to Plant Direct and it would make a useful contribution to plant biology. Thank you for your work.

Author Response:

Plant Direct
Open Access

Detailed response to reviewers' comments: Tanner et al.

Reviewer#1:

This is an interesting study on the subcellular localization of hordein in developing barley endosperm via both immubofluorescent and immunogold-TEM approaches, in which HPF was used to fix the samples for TEM analysis. The quality of most TEM images is acceptable. Overall, the TEM data may provide some useful information for future research in the field.

The speculation "one pathway directly via exocytosis from ER lumen" may not be correct, as "exocytosis" refers to "reaching outside of the cell", rather, the authors may refer "ER protein export" either via COPII vesicles or direct ER to PSV pathway.

Other suggestions for improvement:

1. The term "exocytosis or endocytosed" used in the MS needs to be revised according to the current knowledge in the field (e.g. endocytosis, exocytosis, invagination, engulfment, transport vesicles, membrane fusion).

Response: We must disagree. Export of proteins from plant ER has often been referred to as exocytosis in papers and reviews (eg van de Meene AM, Doblin MS, Bacic A. 2017. The plant secretory pathway seen through the lens of the cell wall. Protoplasma 254, 75-94.) We follow this precedent.

2. Most of the text labelings in the TEM images are too large or not position properly (that mess the structures): better format of arrows should be used.

Response: Agreed. Labels now smaller.

3. Controls for immunogold-TEM should be provided to demonstrate specific labelling in this study.

Response: Agreed. All controls now in Supp. Material: (iLM controls: Sup. Figs. S11 A, B) and iEM controls (Sup. Figs. 12, 13, 14, 15) and noted in methods Line 224, 239 respectively.

4. A discussion on the limitation of fixed samples for iEM should be included vs. live-cell imaging for making conclusions of transport pathways (Figure 13 and title).

Response: Agreed. Discussed Line 537-543. A discussion on the benefits live cell imaging now included eg it would be very helpful to live image the transport process from beginning to end in a single tissue, BUT it is very difficult to transform barley - only one cultivar, Golden Promise, is transformable and transforming that cv involves a large body of work over years with multiple

protein constructs to allow full imaging of key compartments, and is well beyond the scope of this manuscript. The same problem exists for wheat where few cvs are transformable. This is something that may follow from the current study.

5. A summary on the major findings from this study vs. previous studies would be helpful for justifying the novelty of this study.

Response: Agreed. Line 114-115. The existing discussion has been expanded slightly and our input clarified.

6. Some speculations should be backed up by suggestion on future research.
Response: Agreed. Line 529-543. This has been included with live imaging discussion above.

7. Most Figures (2,3,4,7,8,9,10,11) should be modified so that individual images be clearly separated (as shown in Figure 6).

Response: Agreed. All figures have now been separated.

Reviewer#2:

These authors carried out extensive cell biology studies, at both light and electron microscopic levels and also with immunogold labeling, to investigate the processing and packaging of barley endosperm storage proteins. This topic has been investigated frequently in the past in cereals and many papers have been published. The current state of knowledge indicates that cereal seed storage proteins are initially synthesized on ER followed by processing and transport to protein bodies for storage. The manuscript is thoroughly written in the intro and methods sections, but kind of brief in the discussion, which is unfortunate because there appear to be a lot of issues need to be discussed. The authors have presented a good number of micrographs serving as the key experimental evidence in support of the model/process shown in Fig 13. However, the quality (particularly the resolution) of some micrographs is not good enough to be convincing.

While the work does not contain breakthrough results, a couple of unique features are presented:
A. Hordeins and two other storage proteins, serpin Z4 and LTP1, are likely processed in the same route, ending up being stored in vacuoles.
B. The processing of these storage proteins does not traverse the Golgi Apparatus (GA).

While these unique features could further enhance our knowledge in seed storage protein processing and storage, the following points have to be adequately addressed before the

final decision could be made about the suitability of publishing this work in Plant Direct.

1. Two obvious mistakes need to be corrected. First, "LTP" stands for lipid transfer protein, not lipid transferase protein (see Abstract). A name ending with "-ase" is reserved for an enzyme, such as amylase, nuclease, kinase, polymerase, etc,, and lipid carrier protein is not an enzyme because it's not involved in the formation and breakage of a covalent bond.

Response: Agreed, this was a typo. Changed to “lipid transfer protein”

Second, hordeins are glycosylated (for example, see Snegaroff et al (2013) BBA, 1834: 395-403). Thus, the last sentence in the Abstract is erroneous at least for gamma-hordeins.

Response: The omission of Snegarof was a regrettable error. A fulsome discussion of Snagarof and the likely transit of gamma-3 hor through the GA is now included, L 457-497, and additional points as below. We maintain that our iEM shows that at least the dominant B-and C-hordeins do not label GA.

It is clear from Snegarof that gamma-hordein is glycosylated with a xylose and may therefore have transited the GA.

So why don’t we see a signal in the GA using anti-hordein primary antibody ??

1) It may be that although the antibody used here is capable of identifying gamma-1, -2, -3-hordeins on a western blot (Sup Fig S3) that it may not identify gamma-1-hordein in an immuno-stained section. This area needs further work to determine the location gamma-1 hordein.

2) Prof. Michelle Colgrave (Prof. Food and Agriculture, Edith Cowan University, m.colgrave@ecu.edu.au) did an MSMS analysis of hordeins looking for evidence of glycosylation. She said*: “We have searched for glycosylated hordeins by MSMS, gamma-3-hordein in particular in Riso56 and also other lines, looking for evidence of glycosylated hordeins based on modifications commonly observed, eg. glycan residue mass of 162 (hexose sugar) or 203 (HexNAc sugar) or 365 (Hex + HexNAc).We found no evidence of glycosylation, but our studies were not aimed at glyco enrichment and/or analysis. It is quite possible, even probable, that glycopeptides were missed. In this reference (Snegaroff) they use quite specific methods and they also used EndoGlu-C (releases the peptide in question to yield a MS-friendly peptide). We did not use EndoGlu-C. As such, our studies using trypsin or chymo would have produced a much longer peptide and we saw evidence of long peptide sequences and also truncated forms of the theoretical peptide). But it would have been technically challenging to detect glyco forms of these very long sequences. So, I can only conclude that our negative evidence is not proof of absence.”*

We have qualified our suggestion in the discussion, that we found no evidence for glycosylation in barley flour.

2. In Fig 3, the authors claimed that small vacuoles were observed (those circled). How could they rule out the possibility that these "small vacuoles" were actually protein bodies?

Response: Line 290-292: We agree that they are protein bodies, since they are similar in size and frequency to PB identified by immuno-LM, immuno-EM. We have amended discussion to identify them as “protein bodies”.

3. In Fig 4, the authors claimed that small protein bodies were arranged along ER (circled). Without observing ER in the same micrograph, how could they draw this conclusion?

Response: We have removed the suggestion that these are aligned along ER.

4. Fig 5 contains a lot of potentially interesting information, yet the quality is not good enough to reveal the details (especially where those white arrows are). More importantly, what are those small vesicles on the right side of this micrography (just to the left of cell walls)?

Response: The small vesicles on the right side of micrograph are protein bodies, similar to those labelled to the left side of micrograph. To make this clear we have added additional arrows labelling PB on right side of micrograph. We were attempting to demonstrate with this figure, the overall composition of a single cell showing the frequency and spatial arrangement of isolated protein bodies and small clumps of protein bodies and large aggregations of protein bodies which have accumulated inside protein storage vacuoles. This is supported by later EM which lack the overall larger scale picture seen here within a single cell. We think that this justifies inclusion of this micrograph.

5. In Fig 6, what are in the circled regions?

Response: The circled regions are accumulating protein bodies that have not yet joined a vacuole. We have labelled both circled areas as (apb) to indicate “accumulating protein bodies” and this was indicated in the Legend. We have clarified this in the discussion. (L325-326). Fig 6 legend reads:
“Fig. 6: Distribution of LTP1 and serpin Z4 by iLM showing accumulating protein bodies (*apb*), aleurone (*al*), vacuole (*vac*) at mid (15 DPA) and late (30 DPA) stages of endosperm development. Position of single protein body carrying respective protein label shown by white arrow.”

6. In Fig 7A, the authors claimed that blue arrows indicated that cytosolic hordeins were being taking up by vacuoles. There are only three gold particles in total in support of this claims. The authors need to provide much more convincing data to substantiate this potentially intriguing process.

Response: Our intention with this figure was to show the overall location of labelled hordeins at a larger scale than shown in Fig 5 but at the EM level. This figure shows all locations identified by later electron micrographs. We have included additional examples of labelled hordeins close to the cytoplasm/ vacuole boundary and an additional Fig. 7B that shows the same location of hordeins approaching the cytoplasm/ vacuole boundary. 7B also shows an additional unlabelled GA.

The legend now states:
Fig. 7. Overall intracellular distribution of hordeins of barley cv Sloop by iEM. (A) At 6 DPA, gold labelled hordeins (white arrows) were either inside protein bodies (*pb*), the main vacuole (*vacuole*) or a smaller vacuole (*small vacuole*). Hordeins were also found in the cytoplasm (*orange arrows*) and inside vacuoles, as clumps or individual labelled proteins (black arrows). Labelled hordein is also seen outside but closely aligned with protein bodies (circled with dots). This process is explored in later Figures. Individual labelled hordeins were seen close to the vacuole membrane/ cytoplasm junction, either still within the cytoplasm or just inside the vacuole membrane (blue arrows). Primary Ab was mouse anti-hordein B4. Secondary Ab anti-mouse-18 nm gold. Scale 500nm; (B) At 6 DPA a Golgi body (*Golgi*, circled) shows no anti-hordein labelling. Background has been slightly lightened, brightness + 20% to enhance the Golgi body. 2nd antibody only controls show one spot per field at this magnification (Sup. Fig S12); Arrow designation is the same as for (A) showing additional examples of labelled hordeins close to the cytoplasm/ vacuole boundary. All scale bars 500 nm. Primary anti-hordein antibody was 23-3. Secondary Ab anti-mouse-18 nm gold.

7. In Fig 7B, the authors claimed that the black arrows indicated where ribosomes were. This is hardly convincing. Please provide micrography with higher magnification to clearly show that these dots are indeed ribosomes (see my evaluations of Fig 11 below).

Response: We have deleted our images showing these proteins associated with the ER and rely on ample biochemical evidence that hordeins are associated with ER arising initially from biochemical localization of hordeins in isolated microsomes (Cameron-Mills V, Ingversen J, Brandt A. 1978. Transfer of in vitro synthesized barley endosperm proteins into the lumen of the endoplasmic reticulum. Carlsberg Research Communications 4, p91-102). We hope that this will focus the manuscript on our key findings.

8. Fig 8 reveals intriguing information, i.e., there were substantial gold particles outside protein bodies, presumably in cytosol. How could this happen, protein body rupturing or via some other mechanisms? It would be important that the authors can provide a semi-quantitative estimation about the percentage of total hordeins being cytosolic vs in protein bodies/vacuoles. If Fig 8 is a good representative figure, almost 50% of hordeins are actually cytosolic, which is quite significant. How could this much hordeins in protein bodies be released? Does this mean a lot of protein bodies are somehow ruptured along the way to final destination? Or, are there other mechanisms can account for the high percentage of

hordeins being in the cytosol? Furthermore, if hordeins are water-insoluble (as indicated in the very first sentence in the Intro section), how could these cytosolic hordeins stay soluble without the protection of a membranous vesicle?

Response: There are many points here. To make the identification of separate areas circled in this micrograph clearer we present two versions of Fig 9 with and without annotation (in Supp. Figures).

1) *The clustering of labelled hordein closely associated with the protein body:*

We have amplified discussion of the hordeins inside and apparently outside the PB (Line 359-373). This phenomenon is seen in several figures, Figures 10, and Sup Figs S4-S10 all obtained from HPF. All sections above were cut from the same block. Importantly the same appearance was shown in sections cut from separate blocks eg Fig. 12B and D obtained by conventional fixation. We maintain that this accumulation of hordeins around the protein body was not due to bursting of the protein body but represents an uptake mechanism captured by HPF and conventional fixation (Line 365-371).

2) *The quantification of hordeins inside protein bodies, groups of protein bodies outside protein body but withing the area of altered composition associated with the protein body, and isolated labelled hordeins in the cytoplasm:*

   We have amplified discussion of this (Line 371-379). Counts of the proportion of hordeins outside but associated with protein bodies, for all images shown in the ms, is indeed close to 40% (31.2-68.0 % (mean $\pm$ SE, 43.3 $\pm$ 4.5) (calculations shown in Sup. Table T2). The agreement between different figures may indicate a biological process captured in one instant in the same tissue.

3) *The appearance of some isolated hordeins in the cytosol within an area of altered appearance surrounding them:* We hypothesise the appearance is due to ER proteins which surround some hordeins (Discussed Line 380-400, and L 427-434, and point 9 below).

We hope these changes makes the conclusions clearer.

9. Also in Fig 8 (now 10): the "membrane surrounding label" on the left half of the figure is not visible to the eyes of this reviewer, hence not convincing.

Response: The suggestion that there was a surrounding membrane was poorly chosen. We have changed the focus of this point to defining a surrounding area of different texture suggesting that something surrounds some labelled hordeins perhaps heat shock proteins acting as chaperones. We have added magnified examples of labelled hordein surrounded and not surrounded by this area of different texture. The inset images have increased contrast and brightness to enhance the appearance. We hope these changes make the surrounding areas more convincing.

Plant Direct
Open Access

10. In Fig ~~10B~~ (now 12B) there so many gold particles on the peripheral of a protein body. Are these hordeins be taken up by the protein body as suggested in Fig. ~~6~~ 7? Frankly, this photo is more impressive than the two blue arrows in Fig. 6.

Response: Additional examples of this phenomena showing similar areas of uptake of labelled proteins is shown in Fig 11B, D and Supp. Figs S4-10.

11. In Fig 11B, it is hard to recognize those structures pointed by the orange arrows as ribosomes. The authors need to provide more convincing micrographs.

Response: We have deleted micrographs showing ER. See this reviewer's point 7.

12. In ~~Fig 12~~ (now Fig 8), the GA indeed looks clear for hordein signals. Can the authors provide 1-2 more photos like this in the supplemental material section in support of the important notion that hordeins do not traverse GA in its processing?

Response: Additional unlabelled GA image now included at Fig 7B.

13. Since serpin Z4 is synthesized without a signal peptide, how could this protein enter ER and then processed with hordeins in the same protein bodies as the authors have suggested?

Response: Given that Serpin Z4 lacks a conventional transit peptide, we are not sure how Serpin Z4 enters the protein bodies – however the immuno-labelling clearly shows that labelling for serpin Z4 is localised within protein bodies, and the same protein bodies that also contain hordein label. We see evidence of this in many images. We therefore think that this is not an artifact but represents a genuine localisation within protein bodies. We have amended the text to reflect the uncertainty of how serpin Z4 is transferred into protein bodies but we remain convinced that Z4 is present inside protein bodies. Z4 has an internal hydrophobic region of 21 amino-acids (residues 36 - 56) that may serve as a signal for targeting the serpin Z4 to the ER. This is discussed Lines 502-518.
